# What do we mean by preconception health and preconception care in research and policy? A systematic review

**DOI:** 10.1093/humupd/dmag005

**Published:** 2026-03-05

**Authors:** Olivia Chingara, Annabelle Sümegi, Susan Logan, Siladitya Bhattacharya, Andrea Woolner

**Affiliations:** Aberdeen Centre for Women’s Health Research, Institute of Applied Health Sciences, School of Medicine, Medical Sciences and Nutrition, University of Aberdeen, Aberdeen, UK; Aberdeen Centre for Women’s Health Research, Institute of Applied Health Sciences, School of Medicine, Medical Sciences and Nutrition, University of Aberdeen, Aberdeen, UK; Grampian Sexual Health, Community Health and Care Village, Aberdeen, UK; Aberdeen Centre for Women’s Health Research, Institute of Applied Health Sciences, School of Medicine, Medical Sciences and Nutrition, University of Aberdeen, Aberdeen, UK; Aberdeen Centre for Women’s Health Research, Institute of Applied Health Sciences, School of Medicine, Medical Sciences and Nutrition, University of Aberdeen, Aberdeen, UK

**Keywords:** preconception health, preconception care, interconception care, prepregnancy care, reproductive health, pregnancy outcomes

## Abstract

**BACKGROUND:**

Preconception health (PCH) is a globally accepted strategy to reduce preventable adverse pregnancy outcomes and ultimately improve the health of the unborn child. Optimal PCH can be achieved through preconception care (PCC), which encompasses the behavioural, biomedical, and social interventions women and couples undertake and/or receive before conception. However, there is a lack of clarity on various aspects of PCH and PCC, such as what constitutes preconception risk factors, what the optimal interventions are, when the preconception period is, and who the overall target population is. Additionally, marginalised groups such as sexual and racial or ethnic minority individuals are routinely excluded from PCH research and PCC interventions. PCH and PCC are topical issues given changing societal norms worldwide, such as delayed childbirth, exponential rises in fertility treatments, and the growing trend of unplanned pregnancies. We hypothesised that the ambiguity surrounding the definition of PCH and PCC may limit their understanding and application to improve pregnancy and childhood outcomes.

**OBJECTIVE AND RATIONALE:**

This is a systematic review of existing definitions of PCH and PCC to understand the commonalities and disparities in definitions and critical components of PCH and PCC, to aid in the development of a comprehensive and globally standardised definition.

**SEARCH METHODS:**

MEDLINE, PubMed, EMBASE, Cochrane Library, CINAHL, Google Scholar, PsychINFO, and Google were searched to identify published studies, guidelines, and public health websites containing definitions of PCH and PCC published between January 1993 and October 2024. No restrictions were placed on language. We searched academic databases, organisational reports, and policy documents to capture the full range of definitions across clinical, health, and policy contexts.

**OUTCOMES:**

The narrative synthesis of 176 publications showed heterogeneity in the definitions of PCH and PCC. The themes developed from the thematic analysis showed that PCC is preventative care which identifies and utilises interventions to manage individuals’ preconception risk factors and aims to improve pregnancy outcomes by optimising the short- and long-term health of potential parents and their children. The analysis also showed that PCH is relevant across the entire reproductive lifespan. PCC was described as a continuum of care that occurs before conception and encompasses the health of all potential parents, not just women.

**WIDER IMPLICATIONS:**

This systematic review found there is a lack of universality in the definitions of PCH and PCC. Current definitions often narrowly focus on women planning pregnancy, which may exclude important demographics such as unintended pregnancies and fathers, and aligned health needs such as contraception in the preconception period. We propose that there is a need for a definition that captures various demographics and emphasises a life-course approach to reproductive health, acknowledging that the preconception period is much wider than only the period in which couples are actively trying to conceive. Congruence between policymakers, researchers, and public health professionals on the definition of PCH and PCC may address research operationalisation and clinical implementation to better assess global uptake and impact.

**REGISTRATION NUMBER:**

CRD42023480536

## Graphical abstract

**Figure:**
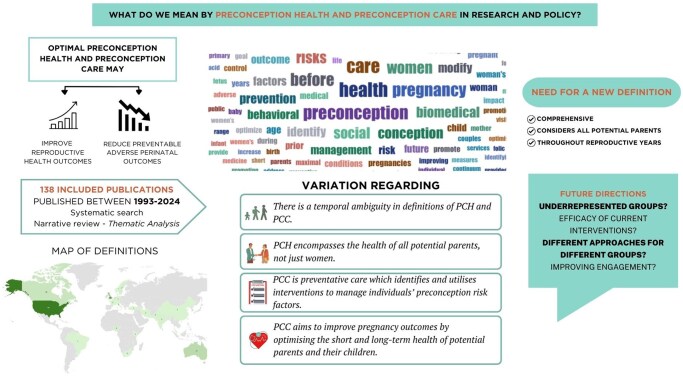
Depiction and overview of the rationale, literature search, methods, and findings of this review on the definitions of preconception health (PCH) and preconception care (PCC).

## Introduction

Global estimates indicate that approximately 800 women and 7700 newborns die every day due to preventable pregnancy and childbirth complications (Chou *et al.*, 2015; World Health Organization, 2024). National and international health organisations such as the Centers for Disease Control and Prevention (CDC) and the World Health Organization (WHO) recognise that optimising preconception health (PCH) and preconception care (PCC) serves as a key strategy to reduce preventable adverse pregnancy and childbirth outcomes such as preterm birth, preeclampsia, and postpartum depression (Poels *et al.*, 2017; Hill *et al.*, 2020). However, the conceptualisation and implementation of PCH and PCC strategies remain inconsistent, and consequently, their impact on pregnancy and childhood outcomes is unclear (Elmir and Schmied, 2022).

PCH refers to an individual’s physical and mental health status and overall wellbeing before conception (World Health Organization, 2013a; Berglund and Lindmark, 2016). Optimal PCH is often hindered by a broad spectrum of health determinants that can influence reproductive outcomes. These include modifiable factors such as nutritional status, body weight, substance use, mental health, chronic disease management, and infectious disease screening, alongside non-modifiable factors including maternal age, genetic predisposition, and ethnicity (Mason *et al.*, 2014; Public Health England, 2018).

The risk factors identified in PCH research, as outlined in Table 1, represent a comprehensive but non-exhaustive list of PCH determinants. Current evidence suggests that most couples have at least one preconception risk factor that may detrimentally affect their birth outcomes (Poels *et al.*, 2017; Public Health England, 2018). Many PCH studies focus on the effect of a single outcome or risk factor, ignoring the effect of multimorbidity on an individual. For example, individuals with diabetes may also have hypertension and obesity. This is particularly problematic as individuals with multimorbidity face an even higher risk of adverse pregnancy outcomes. Furthermore, the increasing prevalence of multimorbidity among individuals of reproductive age highlights the urgent need for more sophisticated approaches to PCH assessment and management. These interconnected health challenges require comprehensive management rather than isolated interventions traditionally addressed through PCC.

**Table 1.** Modifiable preconception risk factors.

Modifiable preconception risk factors

Alcohol use
Environmental/occupational conditions
Gender-based violence
Mental health problems
Nutritional deficiencies
Overweight or obese
Poor nutrition
Pre-existing physical disease
Recreational drug use
Smoking/vaping

PCC encompasses the behavioural, biomedical, and social interventions women, men, and couples receive before conception to improve their health and pregnancy outcomes (World Health Organization, 2013a; Berglund and Lindmark, 2016). These three domains address the biological and lifestyle factors that may pose risks to women, couples, and their children (Mason *et al.*, 2014). Current PCC interventions typically include folic acid supplementation, smoking cessation support, alcohol reduction guidance, vaccination updates, and genetic counselling where appropriate.

However, the implementation of PCC varies considerably across healthcare settings and geographical contexts, creating significant disparities in care delivery. The lack of clear, standardised definitions of PCC makes it challenging to implement consistently and reduces its effectiveness across different healthcare systems. This ambiguity manifests in several ways: the interchangeable use of terms such as PCH, PCC, interpregnancy care, and interconception care by both healthcare professionals and the general public; inconsistent inclusion criteria for PCC interventions; and variation in target populations and timing of interventions (Munthali *et al.*, 2021).

Further, there are substantial gaps between evidence-based risk factors and actual PCC implementation. Studies have shown that although parental mental health and obesity are the most common complications of pregnancy, they are the least likely to be included in PCC interventions (Wilson *et al.*, 2018; Kizirian *et al.*, 2019). The disconnect between evidence and practice highlights the challenges of translating comprehensive PCH research into practical, deliverable care pathways. Healthcare professionals also face multiple barriers to delivering PCC, including time constraints during consultations, insufficient funding, lack of specialised training in PCH assessment, and absence of clear care pathways for complex multimorbidity management (Mazza *et al.*, 2013). This may affect their ability to assess individuals’ PCH and PCC needs adequately.

The timing of PCC interventions also remains debated, as the preconception period is often viewed as the interval between acknowledging intentions to conceive and becoming pregnant (Hill *et al.*, 2020). This perspective fails to acknowledge that approximately 50% of pregnancies are unplanned or mistimed (Lancet, 2018). This pregnancy-intention framework, therefore, excludes approximately half of all pregnancies from benefitting from targeted PCC interventions, creating a significant public health challenge.

The temporal and conceptual relationship between PCH and PCC is fundamental to understanding their interconnected roles in reproductive health. PCH represents the foundational health status that individuals bring to the preconception period, whilst PCC represents the systematic approach to optimising that health status. PCC exists as a necessary response to suboptimal PCH. This nested relationship underscores the importance of comprehensive PCH assessment as a prerequisite for effective PCC delivery.

Despite the array of global policies and initiatives proposed to improve PCH and the increase in publications about PCH and PCC, particularly over the last decade, no published reviews have explored how these terms are defined across policy and research publications. This represents a critical knowledge gap that undermines efforts to develop evidence-based, standardised approaches to preconception health optimisation.

This systematic review aims to address this gap by summarising and analysing PCH and PCC definitions from published studies, relevant guidelines, and public health websites published between 1993 and 2024.

## Methods

### Search strategy

This systematic review examines the research question: What do we mean by PCH and PCC in research and policy? This review was prospectively registered on the PROSPERO International prospective register of systematic reviews (Registration number: CRD42023480536).

MEDLINE, PubMed, EMBASE, Cochrane Library, CINAHL, PsychINFO, and Google Scholar were searched using relevant subject headings, keywords, MeSH headings, and Boolean operators to identify studies containing definitions of PCH and PCC. The search was conducted from January 2024 to December 2024, with the final search being conducted on the 10th of December 2024. The search strategy was developed in consultation with a medical librarian. The search terms used were: ‘preconception health’, ‘preconception care’, ‘pre-conception health’, ‘preconception’, ‘interconception care’, ‘interconception health’, ‘interpregnancy care’, ‘interpregnancy health’, ‘periconception’, ‘prepregnancy care’, ‘pre-pregnancy health’, ‘preconcept*’, ‘pre-concept*’, ‘interconcept*’, ‘inter-concept*’, ‘periconception*’, ‘preconcept$’, ‘pre-concept$’.

Additional resources included bibliographies from relevant articles, personal communication with experts in the field, Google^TM^, clinical guideline websites, and professional organisations. The clinical guideline websites and professional organisations searched are shown in Table 2.

**Table 2.** List of clinical guidelines and professional organisations searched.

Clinical guidelines and professional organisations

American College of Obstetricians and Gynecologists (ACOG)
Royal Australian and New Zealand College of Obstetricians and Gynaecologists (RANZCOG)
Royal Australian College of General Practitioners (RACGP)
Royal College of Obstetricians and Gynaecologists (RCOG)
World Health Organization
Geneva Foundation for Medical Education and Research—Obstetrics and Gynaecology Guidelines
National Institute for Health and Care Excellence (NICE)NHMRC Guidelines Portal

The diverse publication types were chosen as the aim of this review was to capture how PCH and PCC are defined across different contexts. Academic research would provide insights into theoretical foundations and evidence-based definitions, and clinical guidelines and policy documents would reflect how PCH and PCC are defined in practice and healthcare delivery frameworks, whilst health webpages would contain definitions that reflect public understandings.

### Selection process

Publications were included in this review if they were research studies or reviews, clinical policy reports or guidelines, and health web pages where a definition of PCH or PCC or recommendations for population-based PCH or PCC had been provided and published between 1993 and 2024. The latter criterion was included as PCH and PCC are relatively new terms and definitions prior to the last 30 years may no longer be relevant. No restrictions were placed on language or country of publication. Publications exclusively related to antenatal care, pregnancy care, or infertility treatments were excluded.

Two reviewers independently searched, screened the titles and abstracts, identified and reviewed suitable publications, and extracted the relevant data. Any disagreement was resolved by consensus and discussion with the other research team members. Bespoke data extraction tables were used to collect data on study design, setting, publication date, publication type, and definitions of PCH or PCC.

### Data analysis

The review’s primary outcome was to determine the criteria/terminology used to define PCH and PCC. A narrative synthesis using thematic analysis was used to summarise and describe the findings, focusing on the definitions of PCH and PCC (Popay *et al.*, 2006). Data-driven thematic analysis was used to synthesise the definitions into descriptive categories using a semi-grammatical coding method. Thematic analysis is ‘a method for identifying, analysing, and reporting patterns within data’ (Braun and Clarke, 2006). Employing thematic analysis in narrative reviews includes line-by-line coding of the data extracted from publications and generating descriptive codes. The thematic synthesis collated the existing literature to examine how PCH and PCC are viewed, described, and defined in different geographical locations and how definitions of these terms have changed over time. The key findings were organised thematically using NVivo 14, an analytical software that facilitates virtual manual coding and the development of themes (Elliott-Mainwaring, 2021). The thematic synthesis compared definitions across publications to identify common elements that could inform universal definitions of PCH and PCC.

## Results

### Summary

**Figure 1.**
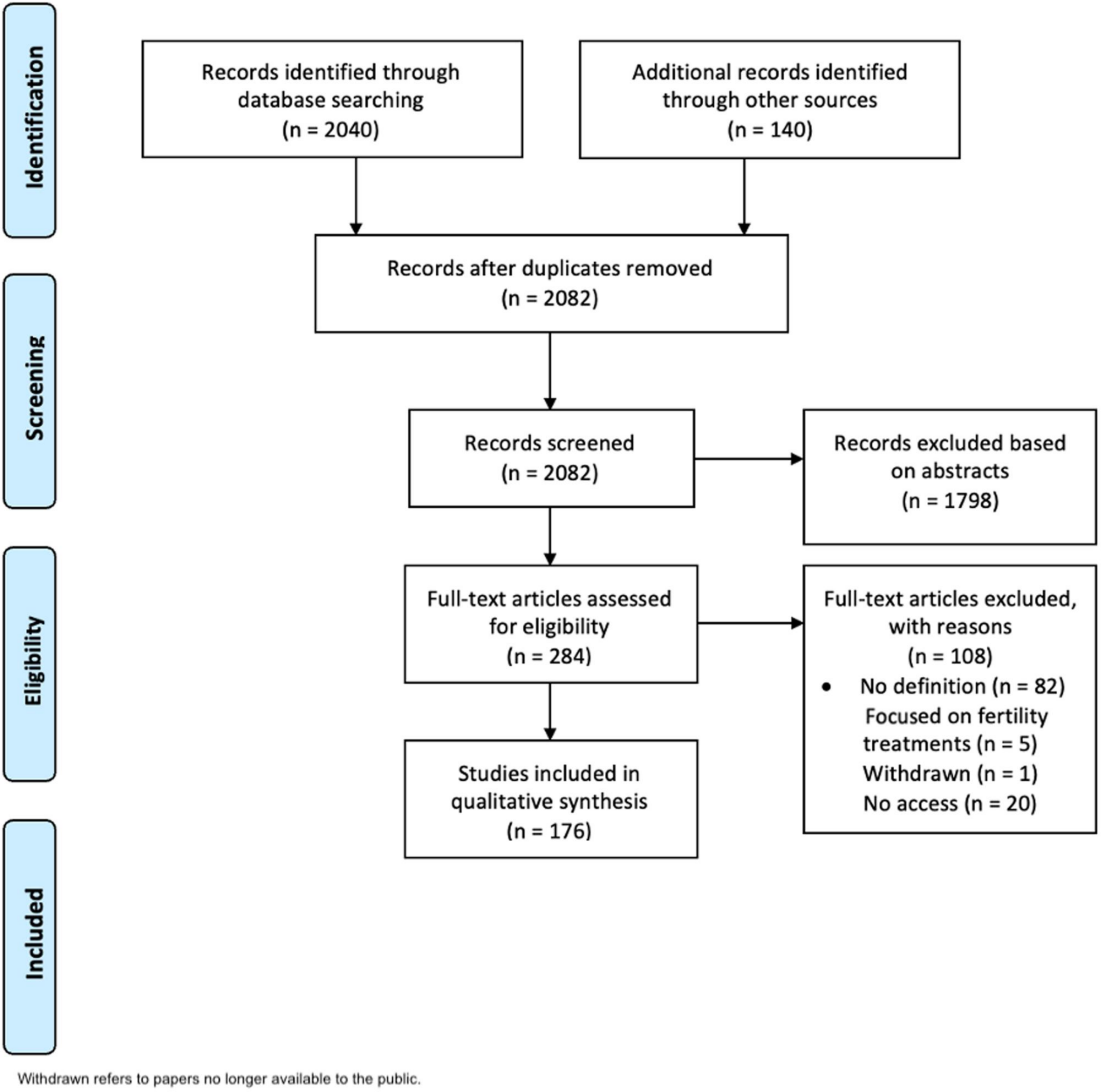
PRISMA flowchart.

The search returned 2180 records (see Fig. 1). A total of 284 full-text records were assessed for eligibility, and 176 studies, published guidelines, and articles from relevant health websites with definitions of preconception health (PCH) and preconception care (PCC) were eligible for inclusion.

### Types of publications

Included were 47 review articles, 39 health webpages, 8 reports, 7 recommendations, 4 supplements, 3 commentaries, 3 expert meetings, 2 consensus statements, two guidelines, one framework, one policy brief, one poster, one consortium, and one book. Additionally, nine systematic reviews, one scoping review, one narrative review, one review protocol, one study protocol, and a range of original research articles (43), using mixed (12), quantitative (21), and qualitative (10) methodology, respectively, were identified.

### Geographical distribution

**Figure 2.**
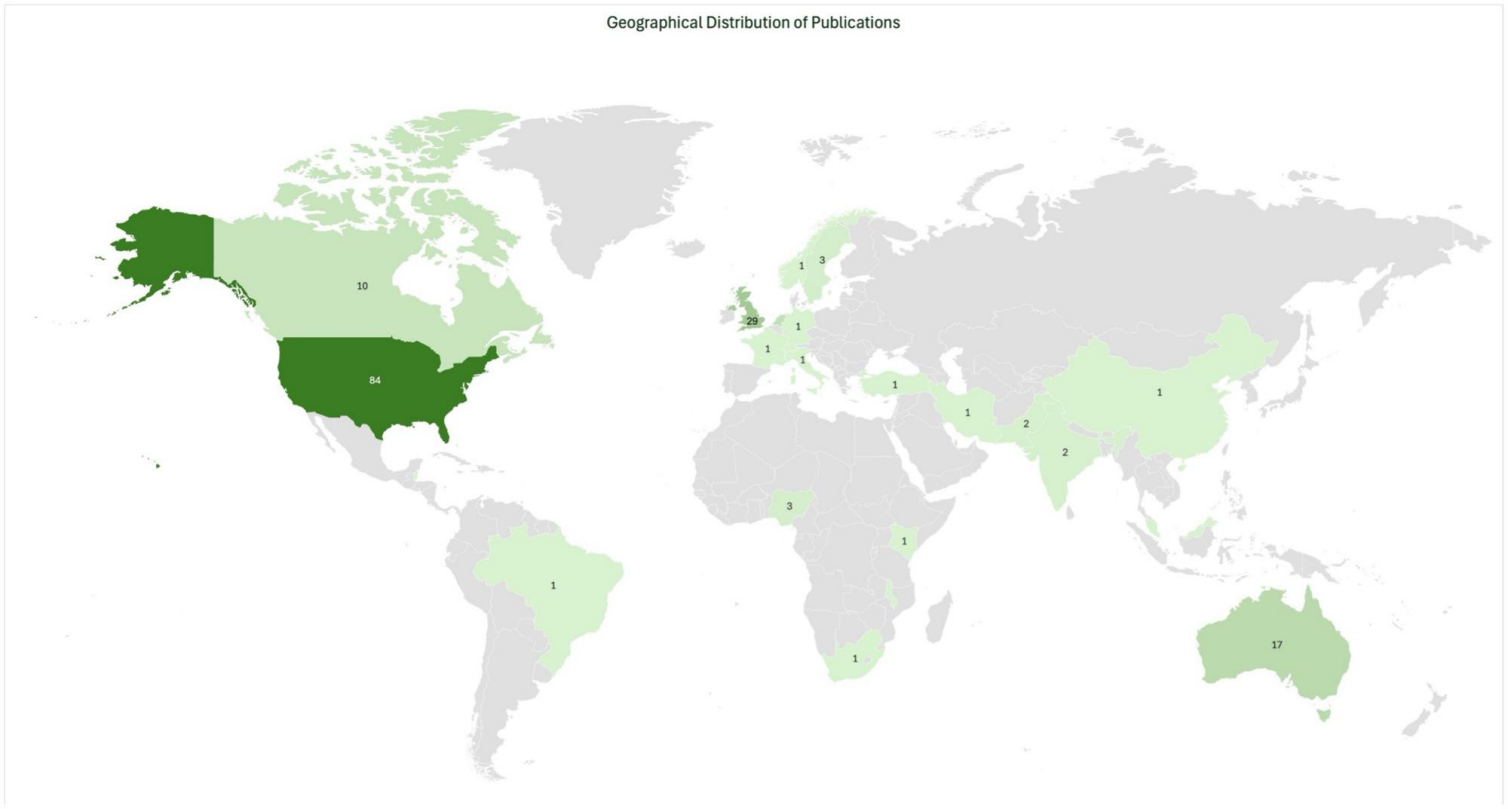
Summary of geographical distribution of publications.

The geographic distribution of the publications included in this review is shown in Fig. 2. Most publications were authored in the United States (84), followed by the United Kingdom (29), Australia (17), and the Netherlands (12). Publications were also authored in Canada (10), India (2), Nigeria (3), Pakistan (2), Sweden (3), Belize (1), Brazil (1), China (1), France (1), Turkey (1), Switzerland (1), Iran (1), Germany (1), Malaysia (1), Malawi (1), Kenya (1), Italy (1), Norway (1), and South Africa (1).

### Year of publication

**Figure 3.**
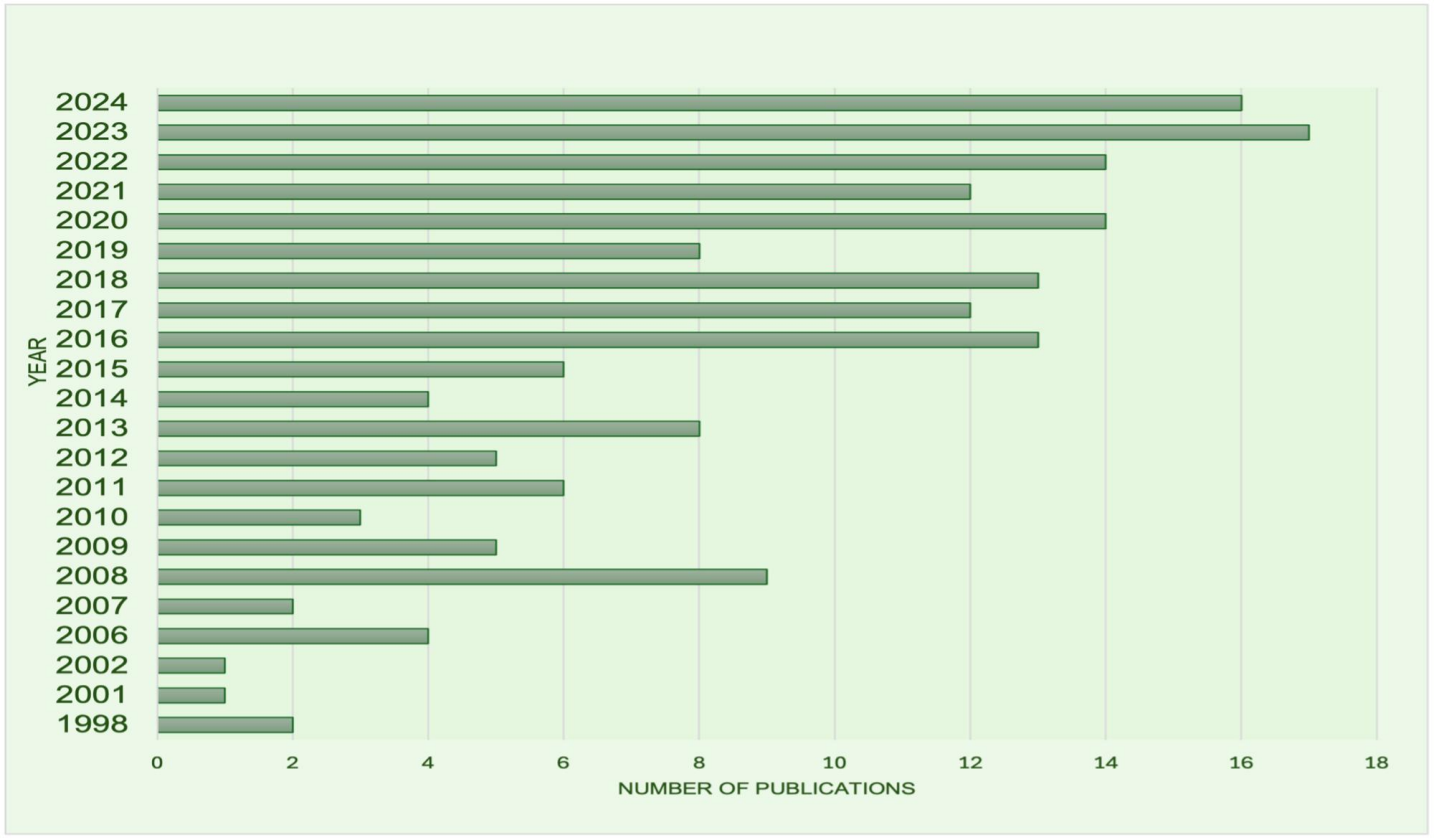
Frequency of publication year.

This review included articles published between 1993 and 2024. The range of publication years is summarised in Fig. 3.

### Conceptual terminology

This review revealed similarities and variations in the definitions of PCH and PCC. These definitions varied in length and complexity, ranging from brief, one-sentence descriptions, for example, ‘care before conception’, to more elaborate definitions spanning several paragraphs. The terms PCH, PCC, pre-pregnancy care (PPC), and interconception care (ICC) were used as separate concepts in many publications; however, they were mostly used interchangeably.

There were 40 publications which distinctly defined PCH (County of Los Angeles, 2001; Feldkamp and Botto, 2008; Johnson *et al.*, 2008; Xaverius *et al.*, 2009; Moos, 2010; Delissaint and McKyer, 2011; Dennis, 2011; Ontario Public Health Association, 2014; Liu, 2015; Berglund and Lindmark, 2016; Nobles‐Botkin *et al.*, 2016; Robbins *et al.*, 2017; Boutain *et al.*, 2018; Harville *et al.*, 2019; Mello *et al.*, 2019; Black *et al*., 2020; Hill *et al.*, 2020; March of Dimes, 2020; Tarasoff *et al.*, 2020; Alabama PH, 2021; Global Foundation for the Care of Newborn Infants (GFCNI), 2020; New Road Medical Centre, 2021; Stanhope and Kramer, 2021; Usman and Mullins, 2021; West of Scotland Managed Clinical Network for Sexual Health Clinical Guidelines Group, 2021; Anakwe *et al.*, 2022; Brooks *et al.*, 2022; Cassinelli *et al.*, 2022; Zaçe *et al.*, 2022; Boyle *et al.*, 2022; Black *et al.*, 2023; Government of Canada, 2023; Verbiest *et al.*, 2025; Algoma PH, 2024; Cassinelli *et al.*, 2024; City of Toronto, 2024; O’Connor *et al.*, 2024; Righton *et al.*, 2024; Rural Health Information Hub, 2024; State of Rhode Island Department, of Health, 2024), nine publications which had definitions of prepregnancy care (Berghella *et al.*, 2010; Hemsing *et al.*, 2017; Firoz *et al.*, 2018; American Society for Reproductive Medicine and American College of Obstetricians and Gynecologists’ Committee on Gynecologic Practice, 2019; Taher SWb, 2019; Marshall and Britton, 2020; The Royal Australian and New Zealand College of Obstetricians, and Gynaecologists, 2024; American College of Obstetricians and Gynecologists, 2024; Parenthood, 2024), and 15 publications which referred to ICC (Johnson *et al.*, 2006; Lu *et al.*, 2006; Atrash *et al.*, 2008; Bish *et al.*, 2012; World Health Organization, 2012; Johnson and Gee, 2015; Huberty *et al.*, 2017; Public Health Agency of Canada, 2017; Dorney *et al.*, 2020; Gregory *et al.*, 2020; Sijpkens *et al.*, 2020; Paladine *et al.*, 2021; American Academy of FP, 2022; Jacob and Hanson, 2022; Reproductive Health National, 2022). Most of these papers mentioned ICC briefly as a subset of PCC, whereas others described ICC as its own entity. The remaining 112 publications specifically defined PCC.

### Thematic analysis

Through the systematic review and narrative synthesis, cumulative definitions of PCH and PCC were derived to consolidate the existing literature. PCH encompasses the overall health of individuals during their reproductive years (regardless of their gender, sexual orientation, or pregnancy intention) with a focus on women, in particular women who had experienced adverse pregnancy outcomes. PCC was defined as care which seeks to identify and modify biomedical, behavioural and social risks to the health of potential parents and future pregnancy outcomes before conception. The narrative synthesis demonstrated that PCC is achieved through prevention, screening and the management of preconception risk factors. The publications suggested that PCC occurs through a continuum of personalised care before conception. This could be in the months or years before pregnancy, across the reproductive lifespan, or between pregnancies, with an aim to reduce adverse health outcomes for babies, mothers and new parents by improving PCH.

The thematic analysis of the definitions included in the publications resulted in the production of four themes, summarised in Tables 3 and 4. These themes highlighted the differences and similarities between the definitions and showed the gaps which exist in the current definitions of PCH and PCC.

**Table 3.** Themes from the thematic analysis of definitions.

Themes
1. There is a temporal ambiguity in definitions of PCH and PCC.
2. PCH encompasses the health of all potential parents, not just women.
3. PCC is preventative care which identifies and utilises interventions to manage individuals’ preconception risk factors
4. PCC aims to improve pregnancy outcomes by optimising the short and long-term health of potential parents and their children.

**Table 4.** Themes from the thematic analysis of definitions.

Themes
1. PCC is preventative care which identifies and utilises interventions to manage individuals’ preconception risk factors.
2. PCC aims to improve pregnancy outcomes by optimising the short-term and long-term health of potential parents and their children.
3. PCH is an individual’s health status across their reproductive lifespan and PCC is a continuum of care which occurs before conception.
4. PCH encompasses the health of all potential parents, not just women.

#### Theme 1: There is a temporal ambiguity in definitions of PCH and PCC

The included publications demonstrated considerable variation in their descriptions of timing, specifically regarding when PCH is most relevant and when PCC should be delivered. Few publications provided specific timeframes. Most included broad timelines, highlighting the lack of consensus regarding optimal timing for preconception interventions.

**Figure 4.**
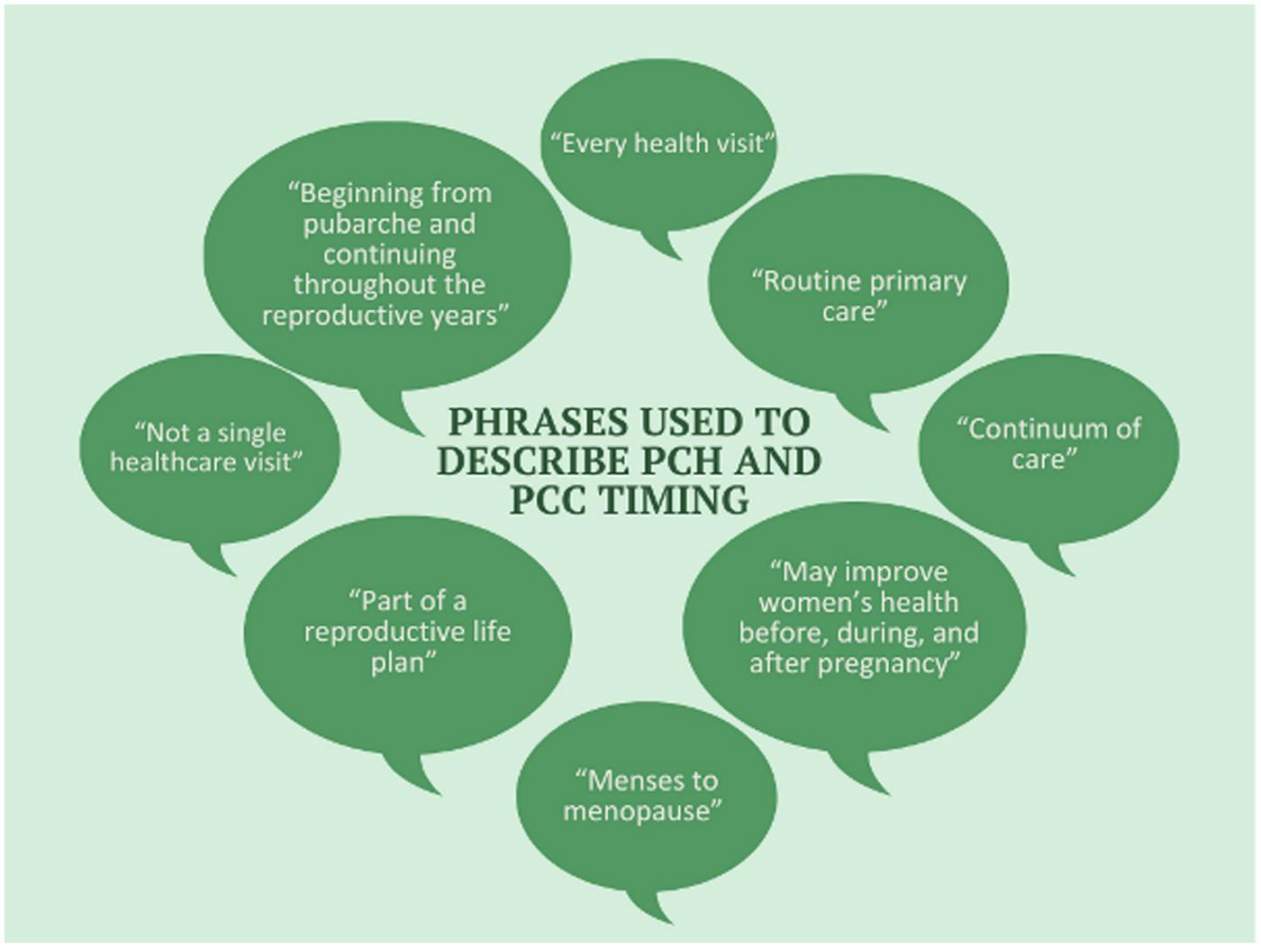
Phrases used to describe preconception health and preconception care timing. PCC: preconception care; PCH: preconception health.

Inconsistencies emerged regarding the timing of PCH relevance. Fourteen definitions indicated that PCH is relevant across the reproductive lifespan (Posner *et al.*, 2006; Dolan *et al.*, 2007; Flenady *et al.*, 2011; Nobles‐Botkin *et al.*, 2016; Hemsing *et al.*, 2017; Public Health Agency of Canada, 2017; Public Health England, 2018; Mello *et al.*, 2019; Paladine *et al.*, 2021; Anakwe *et al.*, 2022; Brooks *et al.*, 2022; Boyle *et al.*, 2022; City of Toronto, 2024; Righton *et al.*, 2024). Figure 4 refers to specific phrases used to describe the timing of PCH across the life course. Conversely, four publications included specific timelines for when PCH is important. Boyle *et al.* (2022) stated that PCH refers to ‘the critical weeks before conception and the period when someone is actively planning to become pregnant’ Lassi *et al.* (2014) and Dean (2013) referred to ‘the period before pregnancy (at least two years) or between consecutive pregnancies’, while Stanhope and Kramer (2021) referred to the “year before pregnancy*”* (Dean *et al.*, 2013; Lassi *et al.*, 2014; Stanhope and Kramer, 2021; Boyle *et al.*, 2022).

A similar temporal variation was observed for PCC, with almost 50% of the included publications concisely referring to PCC as care ‘before conception or pregnancy’ in their definitions (Adams *et al.*, 1993; Akinajo *et al.*, 2019; Allen *et al.*, 2017; Boyle *et al.*, 2022; Callegari *et al.*, 2018; Coonrod *et al.*, 2009; Frayne *et al.*, 2016; Jack *et al.*, 2015; Jacob and Hanson, 2022; Jacob *et al.*, 2019; Kizirian *et al.*, 2019; Mello *et al.*, 2019; Munthali *et al.*, 2021; Nypaver *et al.*, 2016; Ojifinni and Ibisomi, 2020; Price *et al.*, 2019; Righton *et al.*, 2024; Schoenaker, 2024b; St. Fleur *et al.*, 2016; Steegers, 2019; Temel *et al.*, 2015; Thompson *et al.*, 2017; Van Voorst *et al.*, 2017; Verbiest *et al.*, 2025; Vink-van Os *et al.*, 2015; Black *et al.*, 2020; Britton *et al.*, 2023; Dean *et al.*, 2013; Hall *et al.*, 2023; Monson and Jackson, 2016; Peterson *et al.*, 2015; Watterson *et al.*, 2018; Zaçe *et al.*, 2022; Dorney and Black, 2018; Benedetto *et al.*, 2024; World Health Organization, 2012; Wallace and Hurwitz, 1998; Johnson *et al.*, 2006; Farahi and Zolotor, 2013; Xaverius *et al.*, 2009; The Surveillance and Research Workgroup and Clinical Workgroup of the National Preconception Health and Health Care Initiative *et al.*, 2020; Kotelchuck and Lu, 2017; Johnson *et al.*, 2008; Gavin *et al.*, 2014; Schoenaker *et al.*, 2024a; Carter *et al.*, 2023; World Health Organization, 2013a; National Institute for Health and Clinical Excellence, 2023; Shangaris, 2023; New Road Medical Centre, 2021; American College of Obstetricians and Gynecologists, 2024; Government of Canada, 2023; New York State Department of Health, 2013; Public Health Agency of Canada, 2017; University of Monash, 2024; Marshall and Britton, 2020; Malcom, 2018; Rural Health Information Hub, 2024; Dhavliker and Purohit, 2017; Marcin, 2024; Taher SWb, 2019; Watkins, 2023; East Midlands Maternal Medicine Network, NHS England, 2024; Nikolaou, 2023; National Library of Medicine, 2016; Tijoriwala, 2024; Algoma PH, 2024; West of Scotland Managed Clinical Network for Sexual Health Clinical Guidelines Group, 2021; Alabama PH, 2021; European Foundation for the Care of Newborn Infants, 2021; Temel *et al.*, 2014; Okemo *et al.*, 2020). Although these definitions did not indicate a specific time for PCC, a subset of these definitions (9) stipulated that PCC must occur before conception to have a “maximal effect” or “maximal impact” on health outcomes (Johnson *et al.*, 2006; New York State Department of Health, 2013; Temel *et al.*, 2014; Jack *et al.*, 2015; Vink-van Os *et al.*, 2015; Kotelchuck and Lu, 2017; Steegers, 2019; Taher SWb, 2019; Watkins, 2023).

#### Theme 2: PCH encompasses the health of all potential parents, not just women

There was substantial difference in how the publications defined the intended audience for PCH. Whilst women were the most frequently cited demographic, a considerable percentage of the publications recognised the importance of extending PCH considerations to all potential parents regardless of gender or sexual orientation.

**Figure 5.**
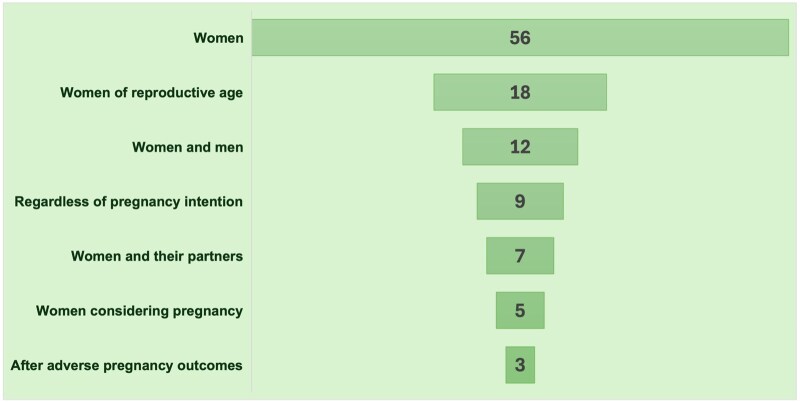
Definitions including women as the target demographic of preconception health.

Most of the publications included in this review detailed the target demographic of PCH. Women were cited the most as the target demographic of PCH, either individually or as a core component, as shown in Fig. 5. Most publications (56) referred to only women as the target audience of PCH (Berghella *et al.*, 2010; Frey *et al.*, 2008; Berglund and Lindmark, 2016; Callegari *et al.*, 2018; Curtis, 2008; Dunlop *et al.*, 2008; Files *et al.*, 2011; Jack *et al.*, 2015; Jacob *et al.*, 2019; Jacob and Hanson, 2022; Liu, 2015; Moos, 2010; Munthali *et al.*, 2021; Nypaver *et al.*, 2016; Paladine *et al.*, 2021; Posner *et al.*, 2006; Robbins *et al.*, 2017; Sardasht *et al.*, 2022; St. Fleur *et al.*, 2016; Steel *et al.*, 2016; Temel *et al.*, 2015; Cheney *et al.*, 2023; Sanders, 2009; Firoz *et al.*, 2018; Feldkamp and Botto, 2008; Flenady *et al.*, 2011; Peterson *et al.*, 2015; Rosenberger *et al.*, 2022; Schoonen *et al.*, 2012; Zaçe *et al.*, 2022; Zühlke and Acquah, 2016; Benedetto *et al.*, 2024; Khekade *et al.*, 2023; World Health Organization, 2012; Wallace and Hurwitz, 1998; Xaverius *et al.*, 2009; Kotelchuck and Lu, 2017; Johnson *et al.*, 2008; Johnson and Gee, 2015; Cassinelli *et al.*, 2024; Carter *et al.*, 2023; American Society for Reproductive Medicine and American College of Obstetricians and Gynecologists’ Committee on Gynecologic Practice, 2019; The Royal Australian and New Zealand College of Obstetricians, and Gynaecologists, 2024; World Health Organization, 2013a; Floyd *et al.*, 2013; Manchester, 2024; Parliament, 2023; New Road Medical Centre, 2021; New York State Department of Health, 2013; Children’s Alliance *et al.*, 2023; Marshall and Britton, 2020; Rural Health Information Hub, 2024; Dhavliker and Purohit, 2017; East Midlands Maternal Medicine Network, NHS England, 2024; Tijoriwala, 2024; Howard *et al.*, 2020). Eighteen publications specifically stated PCH is intended for women of reproductive age (Allaire and Cefalo, 1998; Atrash *et al.*, 2008; Ezegwui *et al.*, 2008; Coonrod *et al.*, 2009; Broussard *et al.*, 2011; Delissaint and McKyer, 2011; Bish *et al.*, 2012; Salihu *et al.*, 2012; Farahi and Zolotor, 2013; Waggoner, 2013; Tydén, 2016; Robbins *et al.*, 2017; Taher SWb, 2019; Ojifinni and Ibisomi, 2020; Pentecost and Meloni, 2020; Alabama PH, 2021; Cassinelli *et al.*, 2022; Santos *et al.*, 2023), five studies noted PCH was for women considering pregnancy (Ezegwui *et al.*, 2008; Edmonds and Ayres, 2017; Usman and Mullins, 2021; American Academy of FP, 2022; Boyle *et al.*, 2022), whilst one publication even noted PCH is for women capable of becoming pregnant (Allaire and Cefalo, 1998). Nine studies stated PCH was necessary regardless of pregnancy intentions or desires (Allaire and Cefalo, 1998; Ontario Public Health Association, 2014; Liu, 2015; New Road Medical Centre, 2021; West of Scotland Managed Clinical Network for Sexual Health Clinical Guidelines Group, 2021; American Academy of FP, 2022; Children’s Alliance *et al.*, 2023; City of Toronto, 2024; Manchester, 2024).

Although women were most commonly referenced as the intended audience of PCH, several studies included other demographics in their definitions. There were 22 publications which listed that PCH was the health of all individuals (Van Der Zee and De Beaufort, 2011; Ontario Public Health Association, 2014; Arluck and Mayhew, 2018; Public Health England, 2018; Sijpkens *et al.*, 2020; Tarasoff *et al.*, 2020; The Surveillance and Research Workgroup and Clinical Workgroup of the National Preconception Health and Health, Care Initiative *et al.*, 2020; European Foundation for the Care of Newborn Infants, 2021; Stanhope and Kramer, 2021; American Academy of FP, 2022; Reproductive Health National, 2022; Hall *et al.*, 2023; Harris *et al.*, 2023; Khekade *et al.*, 2023; Verbiest *et al.*, 2025; Montanaro *et al.*, 2023; Parliament, 2023; Cassinelli *et al.*, 2024; City of Toronto, 2024; Schoenaker *et al.*, 2024a, 2024b), four which referred to patients as the target audience of PCH (Files *et al.*, 2008; Marshall and Britton, 2020; American Academy of FP, 2022; Dude *et al.*, 2022), and 14 which included men in their description of PCH (Frey *et al.*, 2008; Jack *et al.*, 2008; Gavin *et al.*, 2014; Lassi *et al.*, 2014; Nobles‐Botkin *et al.*, 2016; Hemsing *et al.*, 2017; Public Health Agency of Canada, 2017; Thompson *et al.*, 2017; Harville *et al.*, 2019; Mello *et al.*, 2019; Black *et al.*, 2020; West of Scotland Managed Clinical Network for Sexual Health Clinical Guidelines Group, 2021; Anakwe *et al.*, 2022; Government of Canada, 2023). Four publications mentioned sexual orientation or gender in their descriptions of the PCH target demographic (Ontario Public Health Association, 2014; Marshall and Britton, 2020; Montanaro *et al.*, 2023; City of Toronto, 2024). Marshall & Britton (2020) referred to ‘women’ throughout their article but noted that transmen and gender-non-confirming individuals may also become pregnant (Marshall and Britton, 2020).

#### Theme 3: PCC is preventative care which identifies and utilises interventions to manage individuals’ preconception risk factors

The thematic analysis showed PCC is predominantly viewed as the provision of preventative care, management of existing health conditions, and assessment of factors that predispose individuals to adverse reproductive outcomes. There was congruence amongst the definitions that whilst interventions may effectively address modifiable risks, identification and counselling remain crucial for non-modifiable preconception risk factors.

A total of 80 publications referred to PCC as preventative care, which includes the identification and management of preconception risks (Allaire and Cefalo, 1998; Frey, 2002; Johnson *et al.*, 2006; Atrash *et al.*, 2008; Curtis, 2008; Feldkamp and Botto, 2008; Files *et al.*, 2008, 2011; Jack *et al.*, 2008; Coonrod *et al.*, 2009; Sanders, 2009; Xaverius *et al.*, 2009; Berghella *et al.*, 2010; Moos, 2010; Broussard *et al.*, 2011; Delissaint and McKyer, 2011; Van Der Zee *et al.*, 2011; Guler Baysoy and Özkan, 2012; Salihu *et al.*, 2012; World Health Organization, 2012; Dean *et al.*, 2013; Farahi and Zolotor, 2013; Floyd *et al.*, 2013; New York State Department of Health, 2013; Shannon *et al.*, 2013; World Health Organization, 2013a; Ontario Public Health Association, 2014; Temel *et al.*, 2014; Jack *et al.*, 2015; Johnson and Gee, 2015; Liu, 2015; Vink-van Os *et al.*, 2015; Bø *et al.*, 2016; Frayne *et al.*, 2016; St. Fleur *et al.*, 2016; Steel *et al.*, 2016; Tydén, 2016; Zühlke and Acquah, 2016; Dhavliker and Purohit, 2017; Edmonds and Ayres, 2017; Kotelchuck and Lu, 2017; Poels *et al.*, 2017; Public Health Agency of Canada, 2017; Robbins *et al.*, 2017; Thompson *et al.*, 2017; Van Voorst *et al.*, 2017; Boutain *et al.*, 2018; Dorney and Black, 2018; Government of Belize and Ministry of Health, 2018; Malcom, 2018; Simone *et al.*, 2018; Watterson *et al.*, 2018; Akinajo *et al.*, 2019; Jacob *et al.*, 2019; Steegers, 2019; Gregory *et al.*, 2020; Howard *et al.*, 2020; Sijpkens *et al.*, 2020; Tarasoff *et al.*, 2020; Methodist, 2021; Munthali *et al.*, 2021; Murugappan *et al.*, 2021; The Royal Australian and New Zealand College of Obstetricians, and Gynaecologists, 2024; Cassinelli *et al.*, 2022; Dude *et al.*, 2022; Reproductive Health National, 2022; Withanage *et al.*, 2022; Boyle *et al.*, 2022; Khekade *et al.*, 2023; Montanaro *et al.*, 2023; National Institute for Health and Clinical Excellence, 2023; Parliament, 2023; Santos *et al.*, 2023; American College of Obstetricians and Gynecologists., 2024; Benedetto *et al.*, 2024; Cassinelli *et al.*, 2024; Marcin, 2024; Parenthood, 2024; Schoenaker *et al.*, 2024a; University of Monash, 2024). There were 20 publications which spoke specifically about medical problems and the importance of managing health conditions before pregnancy through PCC (Dunlop *et al.*, 2008; Ezegwui *et al.*, 2008; Guler Baysoy and Özkan, 2012; Jack *et al.*, 2015; Johnson and Gee, 2015; Liu, 2015; Berglund and Lindmark, 2016; Thompson *et al.*, 2017; Firoz *et al.*, 2018; Government of Belize and Ministry of Health, 2018; Simone *et al.*, 2018; Dorney *et al.*, 2020; Ojifinni and Ibisomi, 2020; Sijpkens *et al.*, 2020; Zaçe *et al.*, 2022; Khekade *et al.*, 2023; American College of Obstetricians and Gynecologists., 2024; Manchester, 2024; Marcin, 2024; O’Connor *et al.*, 2024). There were also 89 publications which referred to the management of preconception risk factors using interventions, measures or activities (Allaire and Cefalo, 1998; Frey, 2002; Johnson *et al.*, 2006; Atrash *et al.*, 2008; Curtis, 2008; Jack *et al.*, 2008; Coonrod *et al.*, 2009; Sanders, 2009; Xaverius *et al.*, 2009; Berghella *et al.*, 2010; Moos, 2010; Raynal, 2010; Broussard *et al.*, 2011; Delissaint and McKyer, 2011; Files *et al.*, 2011; Flenady *et al.*, 2011; Salihu *et al.*, 2012; Schoonen *et al.*, 2012; World Health Organization, 2012; Dean *et al.*, 2013; Farahi and Zolotor, 2013; Floyd *et al.*, 2013; New York State Department of Health, 2013; World Health Organization, 2013a, 2013b; Ontario Public Health Association, 2014; Jack *et al.*, 2015; Peterson *et al.*, 2015; Vink-van Os *et al.*, 2015; Berglund and Lindmark, 2016; Bø *et al.*, 2016; Nypaver *et al.*, 2016; St. Fleur *et al.*, 2016; Steel *et al.*, 2016; Tydén, 2016; Zühlke and Acquah, 2016; Dhavliker and Purohit, 2017; Kotelchuck and Lu, 2017; Poels *et al.*, 2017; Public Health Agency of Canada, 2017; Robbins *et al.*, 2017; Boutain *et al.*, 2018; Callegari *et al.*, 2018; Dorney and Black, 2018; Government of Belize and Ministry of Health, 2018; Malcom, 2018; Simone *et al.*, 2018; Akinajo *et al.*, 2019; Jacob *et al.*, 2019; Kizirian *et al.*, 2019; Price *et al.*, 2019; Steegers, 2019; Taher SWb, 2019; Black *et al.*, 2020; Howard *et al.*, 2020; Marshall and Britton, 2020; Okemo *et al.*, 2020; Sijpkens *et al.*, 2020; Tarasoff *et al.*, 2020; Methodist, 2021; Munthali *et al.*, 2021; Sijpkens *et al.*, 2021; Stanhope and Kramer, 2021; Brooks *et al.*, 2022; Cassinelli *et al.*, 2022; Jacob and Hanson, 2022; Reproductive Health National, 2022; Rosenberger *et al.*, 2022; Withanage *et al.*, 2022; Zaçe *et al.*, 2022; Boyle *et al.*, 2022; Carter *et al.*, 2023; Children’s Alliance *et al.*, 2023; Khekade *et al.*, 2023; Montanaro *et al.*, 2023; National Institute for Health and Clinical Excellence, 2023; Parliament, 2023; Santos *et al.*, 2023; Verbiest *et al.*, 2025; American College of Obstetricians and Gynecologists., 2024; Benedetto *et al.*, 2024; Cassinelli *et al.*, 2024; O’Connor *et al.*, 2024; Schoenaker *et al.*, 2024a, 2024b; Tijoriwala, 2024; University of Monash, 2024). Moreover, PCC as a ‘set of interventions to identify and modify biomedical, behavioural, and social risks’ was included in most definitions. This is included in the original definition posed by Johnson *et al.* (2006), which was referenced by most included publications (Johnson *et al.*, 2006). Additionally, five publications noted that PCC should be individualised care designed to meet unique needs (Files *et al.*, 2008; Jack *et al.*, 2008; Public Health England, 2018; American Academy of FP, 2022; Manchester, 2024).

#### Theme 4: PCC aims to improve pregnancy outcomes by optimising the short and long term health of potential parents and their children

The narrative synthesis identified health promotion and optimisation as central objectives with PCC definitions, with outcomes extending beyond immediate pregnancy concerns to encompass broader health benefits for potential parent and future children. Multiple domains and timeframes emerged as targets for PCC interventions, reflecting the multifaceted nature of PCC goals.

Health promotion or optimisation was noted as an integral component of PCC. A total of 52 publications included health promotion in their definition of PCC (Wallace and Hurwitz, 1998; County of Los Angeles, 2001; Frey, 2002; Posner *et al.*, 2006; Johnson *et al.*, 2008; Files *et al.*, 2008, 2011; Coonrod *et al.*, 2009; Broussard *et al.*, 2011; Flenady *et al.*, 2011; Bish *et al.*, 2012; Salihu *et al.*, 2012; Schoonen *et al.*, 2012; Farahi and Zolotor, 2013; Shannon *et al.*, 2013; World Health Organization, 2013a; Lassi *et al.*, 2014; Peterson *et al.*, 2015; Monson and Jackson, 2016; Tydén, 2016; Bhutta *et al.*, 2017; Edmonds and Ayres, 2017; Hemsing *et al.*, 2017; Poels *et al.*, 2017; Public Health Agency of Canada, 2017; Van Voorst *et al.*, 2017; Callegari *et al.*, 2018; Dorney and Black, 2018; Public Health England, 2018; American Society for Reproductive Medicine and American College of Obstetricians and Gynecologists’ Committee on Gynecologic Practice, 2019; Black *et al.*, 2020; Dorney *et al.*, 2020; Howard *et al.*, 2020; Okemo *et al.*, 2020; Pentecost and Meloni, 2020; Margerison *et al.*, 2021; Methodist, 2021; Murugappan *et al.*, 2021; Cassinelli *et al.*, 2022; Rosenberger *et al.*, 2022; Sardasht *et al.*, 2022; Children’s Alliance *et al.*, 2023; Harris *et al.*, 2023; Khekade *et al.*, 2023; Montanaro *et al.*, 2023; American College of Obstetricians and Gynecologists, 2024; Benedetto *et al.*, 2024; Cassinelli *et al.*, 2024; City of Toronto, 2024; Marcin, 2024; Righton *et al.*, 2024; Rural Health Information Hub, 2024). The publications included a myriad of goals that PCC seeks to achieve, such as improved women’s health and pregnancy outcomes and short-term and long-term health, as shown in Table 5.

**Table 5.** Goals of preconception care.

Preconception care goals	Publications
Women’s health and pregnancy outcome	61
Future pregnancies or children	41
Health of expectant parents	16
Increase PCH and PCC knowledge	14
Short-term and long-term health	12
Foetal health	9
Increase in planned pregnancies	2

## Discussion

### Principal findings

This systematic review highlighted that the current landscape of preconception health (PCH) and preconception care (PCC) definitions is characterised by fundamental inconsistencies that crucially undermine effective implementation and evidence synthesis. Beyond the absence of a universally accepted definition, this analysis of 176 publications demonstrates that existing definitions of PCH and PCC fail to adequately address contemporary reproductive health challenges, including the diverse needs of all potential parents, the complexity of timing consideration, and the multifaceted nature of preconception risk factors. The findings of this systematic review has implications for clinical practice, public health policy and research gaps in reproductive health.

The thematic analysis identified four key themes which captured the commonalities and disagreements and ambiguity that exists within definitions of PCH and PCC. First, the marked variation in timing specifications, such as “critical week” and “across the reproductive lifespan” creates confusion about optimal intervention periods and hinders the implementation of PCC. Second, the persistent focus on women of reproductive age, despite growing recognition of male/partner contributions to pregnancy outcomes, perpetuates gender-biased approaches to reproductive health. Third, whilst most definitions acknowledge the importance of identifying and managing preconception risk factors, there is insufficient recognition of broader environmental exposures, systemic inequalities and structural determinants that influence reproductive outcomes. Finally, the goals of PCC remain poorly articulated, with limited consideration of how interventions should differ based on pregnancy intentions, contraceptive use and diverse family-building approaches.

The definitional limitations have cascading effects on various aspects of public health. It creates barriers to effective communication between healthcare professionals, policymakers, and the public, and fails to address how PCC should respond to contemporary reproductive realities, including high rates of unintended pregnancy, falling birth rates, diverse family structures, and persistent health inequalities.

### Meaning of the results

The evolution of PCH and PCC definitions over three decades reflects both progress and stagnation in reproductive health understandings. Despite the Centers for Disease Control and Prevention’s (CDC) landmark publication in 2006, “Recommendations to Improve Preconception Health and Healthcare”, which included ten recommendations with an increased focus on PCH, there has been a relatively low publication rate in this area since (Johnson *et al.*, 2006). Notably, the CDC definition was referenced by many publications included in this review. Whilst significant, these recommendations are now 18 years old, and many of the reproductive challenges faced globally, such as unplanned pregnancies, falling birth rates and use of assisted reproduction, have increased (Lancet, 2018).

The evolution of the definitions from detailed explanations to more concise descriptions support attempts to make definitions of PCH and PCC more accessible and communicable. Conversely, it may also indicate that original definitions no longer address contemporary reproductive health issues and are not fit for purpose. This evolution may have inadvertently obscured essential complexities of PCH and PCC that must be addressed for effective implementation. For example, this review highlighted the importance of the semantics of the language used and how it may exclude groups of people and consequently impact health outcomes and the delivery of care. This mirrors the findings of a recent public consultation, which shows that the language used to describe PCH may be limiting its implementation and improvement (Schoenaker *et al.*, 2024a).

The persistent emphasis on individual-level interventions, whilst important, inadequately addresses structural determinants of reproductive health outcomes. Exposure to systemic racism, socioeconomic disadvantage and environmental hazards profoundly influence preconception health, yet current definitions provide limited guidance on how PCC should address these broader determinants. Additionally, this individualistic approach may inadvertently perpetuate health inequalities by placing responsibility for complex, multifactorial outcomes primarily on individuals rather than addressing systemic barriers.

The minimal attention to contraception, pregnancy planning and reproductive autonomy within current definitions represents a significant oversight. Given that over 50% of pregnancies are unintended, PCC approaches would benefit from integrating reproductive choice and contraceptive counselling as core components rather than peripheral consideration. Furthermore, the relationship between PCC and reproductive rights requires careful navigation to ensure that health promotion does not become prescriptive or paternalistic, particularly for marginalised populations who have historically experienced reproductive coercion.

### Strengths and limitations

This review had various strengths. To our knowledge, this is the first review to analyse PCH and PCC definitions in research studies, clinical guidelines and policies. The selection criteria were developed to include PCH and PCC definitions from various professional stakeholder publications, including published research, reports, guidelines, and health web pages. This enabled a diverse and multifaceted review of the definitions. Another strength is the systematic review design, with a narrative thematic synthesis, enabling a broader scope of the literature review whilst following an explicit pre-specified methodology (Turnbull *et al.*, 2023).

Several limitations warrant consideration. Due to the nature of thematic analysis, researcher bias may have impacted the findings, as is possible in any qualitative analysis. To mitigate this potential limitation, we utilised bracketing to produce a data-driven analysis (Tufford and Newman, 2012). The geographical distribution of publications is a limitation, as the majority originated from high-income countries. This may limit the generalisability of the findings to low and middle-income countries (LMICs), though it highlights the disparity of PCH and PCC publications, globally. LMICs have a higher burden of adverse pregnancy outcomes such as maternal and neonatal mortality and may require differing PCH and PCC approaches due to variations in disease burden and preconception risk factors (Young *et al.*, 2013). Whilst no restrictions were placed on the language of the publications, the search only resulted in one non-English publication. This may have been affected by the Google^TM^ search engine built-in filter, which adjusts results based on location. We were unable to access 20 publications which may represent a potential source of bias in our findings.

### Comparison with literature

A key finding was the variability in how different publications approached the timing of PCH and when the delivery of PCC interventions should occur. In their Lancet series on PCH, Stephenson *et al.* conceptualised the preconception period using three perspectives: biological health (days to weeks prior to embryo development), individual health (weeks to months of conscious intention to conceive), and public health (months to years before conception) (Stephenson *et al.*, 2018). Although the wider literature has indicated the time sensitivity of the preconception period, the timing of PCH and PCC was vaguely described as “before conception” in this review (Welshman *et al.*, 2023). This oversimplification may impede the effective delivery of interventions, particularly for preconception risk factors like obesity, which require extended periods for modification and management (Harville *et al.*, 2019). The high prevalence of unplanned pregnancies underscores the importance of early intervention strategies and a life course approach (Lancet, 2018). Several publications in this review explicitly stated the relevance of PCH and PCC across the reproductive lifespan. To achieve behavioural changes at a population level, PCH education may need to begin earlier, during school (Schoenaker *et al.*, 2024a).

Most publications referred to women of reproductive age as the target demographic of PCH. The emphasis on women of reproductive age places the onus of pregnancy outcomes on women and ignores the contributions of men/partners, other family members, friends and the wider public, who may influence decisions and views on reproductive health and future pregnancy planning both societally and for individuals. This stance also reinforces societal expectations on women regarding reproduction, including contraceptive use and preventing pregnancy (Kimport, 2018; Welshman *et al.*, 2023). Other descriptors of the target demographic of PCH were women capable of becoming pregnant and women considering pregnancy. This assumes all women can become pregnant, despite research showing that fertility issues affect over 10.43 million women aged 15 to 34 years who are trying to conceive (Nugent and Chandra, 2024). Furthermore, positioning PCH as exclusively for women considering pregnancy alienates women who have unintended pregnancies, which account for over 50% of pregnancies (Lancet, 2018) and women who may not perceive themselves as a “future parent”. It also contributes to the class disparities observed in adverse pregnancy outcomes, as indicated by studies showing that lower socioeconomic status and younger age are independent risk factors for unplanned pregnancies (Iseyemi *et al.*, 2017).

Notably, only four publications referred to sexual orientation and gender in their definitions. LGBTQIA+ individuals, specifically lesbian and bisexual women, are more likely to have unintended pregnancies and poor PCH (Stroumsa and Johnson, 2020). Excluding diverse populations from definitions of PCH may promote the idea that it is not intended for them, which may limit their care-seeking behaviour. It also affects care guidelines and medical training as a lack of inclusion in guidelines further limits the ability of care professionals to be adequately prepared to provide PCC to diverse audiences. Research has shown that healthcare professionals often have limited knowledge about parenting pathways for LGBTQIA+ families and the ability to provide gender-affirming care (Permezel *et al.*, 2023). Further, a study of healthcare teaching in UK medical schools showed that only 40.9% of students reported being taught about LGBTQ+ healthcare (Barber *et al.*, 2023).

This review revealed several key findings regarding the definitions of PCH and PCC. Throughout the definitions, PCC as preventative care was the most similar finding between the publications. Within the preventative care theme, managing health conditions was explicitly described by 20 publications. Although pre-existing health conditions such as diabetes can significantly impact maternal and foetal outcomes, framing PCC exclusively as care for individuals with health problems may inadvertently discourage engagement from those who do not perceive themselves as ill (Van Der Zee *et al.*, 2013). Whether PCH and PCC policies specifically target those with recognised conditions requiring optimisation or the wider public, including “healthy” individuals, needs to be clear.

### Clinical implications

The findings of this review have various implications for how PCC is delivered across multiple healthcare settings. Rather than limiting discussions about PCH to women’s reproductive health appointments, it would be beneficial to develop integrated approaches that engage all potential parents across diverse clinical encounters. This requires expanding beyond traditional reproductive health services to include primary care, mental health and substance abuse treatment, and specialist medical services. Clinical services must be adapted to address the needs of individuals across the spectrum of pregnancy intentions, ranging from those actively planning pregnancy to those at risk of unintended pregnancy and those who do not intend to conceive. This necessitates nuanced approaches that respect reproductive autonomy whilst providing appropriate health information and interventions.

The PCH and PCC needs of individuals of diverse gender identities, sexual orientations and cultural backgrounds should be considered. This means there is a need for further research to identify the needs of specific groups and the development of additional training pathways for healthcare professionals. This includes developing competency in caring for LGBTQIA+ individuals, single parents by choice and families formed through assisted reproductive technologies.

Electronic health records should be optimised to support PCH and PCC by incorporating risk screening tools, clear referral pathways, and follow-up appointments. This may mitigate current and future strains on the National Health Service (NHS) and public health due to unplanned pregnancies. Additionally, considering the current limitations of the NHS, clinicians may need to consider different approaches to PCC. Healthcare professionals need to be equipped to discuss how reproductive life planning intersects with health optimisation, ensuring that individuals can make informed decisions about if when and, how to become pregnant whilst maintaining optimal health throughout their reproductive years. Professionals should be prepared to use novel approaches such as social media and popular figures to spread public health messages.

### Research implications

This review establishes a foundation for developing evidence-based, inclusive definitions of PCH an PCC, but achieving this goal requires coordinated action beyond traditional research methods. We propose a multi-phase approach, beginning with expert consensus processes that utilise these findings to develop preliminary frameworks for updated definitions. These frameworks should explicitly address timing considerations, target populations, intervention scope and outcome measures whilst incorporating perspectives from diverse stakeholders including healthcare professionals, public health experts, community representatives and individuals with lived experience of reproductive healthcare.

The research agenda must prioritise understanding how environmental and structural factors should be incorporated into PCH and PCC approaches. This includes investigating how exposure to systemic racism, ableism, environmental toxins and socioeconomic disadvantage should inform individual and population-level interventions. Research is needed on how PCC can address intergenerational transmission of health risks whilst acknowledging that individual-level interventions may be insufficient to address structurally determined health outcomes.

Investigating the perceptions and needs of men, same-sex couples, non-binary individuals, and people with disabilities may develop targeted interventions to improve reproductive health outcomes. Large-scale studies are needed to evaluate the most effective PCC interventions and how PCC is best delivered for these diverse populations. For example, is it more effective to provide interventions for men’s PCH through individual care or are male-focused interventions better integrated with couple-based or family-centred approaches.

## Conclusion

The absence of clear, inclusive and evidence-based definitions of PCH and PCC represents a fundamental barrier to advancing reproductive health outcomes globally. This systematic review demonstrates that current definitional inconsistencies have consequences for research quality, clinical practice and health equity. The findings show that existing approaches inadequately address contemporary reproductive realities and fail to incorporate essential considerations about timing, target populations, intervention scope and implementation contexts. Definitions of PCH and PCC need to evolve from the narrow focus on women planning pregnancy to a contemporary, more comprehensive, life-course and population-level approach to PCH. PCH applies to all potential parents, including men, same-sex couples and individuals across the gender spectrum; thus, the PCH of all potential parents should be considered whilst being mindful that many people in society have no intention of becoming parents. Optimal PCH and PCC are critical strategies for improving individual and population health outcomes. We conclude that these outcomes relating to preconception will not substantially improve without universally accepted definitions of PCH and PCC and an evidence base to support interventions and recommendations.

## Data Availability

The data underlying this article will be shared, where possible, on reasonable request to the corresponding author.

## References

[dmag005-B1] Adams MM , Bruce FC, Shulman HB, Kendrick JS, Brogan DJ. Pregnancy planning and pre-conception counseling. The PRAMS Working Group. Obstet Gynecol 1993;6:955–959. 8233272

[dmag005-B2] Akinajo O , Osanyin G, Okojie O. Preconception care: assessing the level of awareness, knowledge and practice amongst pregnant women in a tertiary facility. J Clin Sci 2019;16:87.

[dmag005-B3] Alabama PH. Preconception health. 2021.

[dmag005-B4] Algoma PH. Preconception health. 2024.

[dmag005-B5] Allaire AD , Cefalo RC. Preconceptional health care model. Eur J Obstet Gynecol Reprod Biol 1998;78:163–168. 9622313 10.1016/s0301-2115(98)00062-1

[dmag005-B6] Allen D , Hunter MS, Wood S, Beeson T. One Key Question^®^: first things first in reproductive health. Matern Child Health J 2017;21:387–392. 28220337 10.1007/s10995-017-2283-2

[dmag005-B7] American Academy of FP. Preconception care (Position Paper). 2022.

[dmag005-B8] American College of Obstetricians and Gynecologists. Good health before pregnancy: prepregnancy care. 2024.

[dmag005-B9] American Society for Reproductive Medicine, American College of Obstetricians and Gynecologists’ Committee on Gynecologic Practice. Prepregnancy Counseling. Fertil Steril 2019;1:32–42. 10.1016/j.fertnstert.2018.12.003 30611411

[dmag005-B10] Anakwe A , BeLue R, Xian H, Xaverius P. Men’s preconception health and fertility intentions: a latent class analysis approach. 2022;6:155798832211357. 10.1177/15579883221135764 PMC9663665 36373425

[dmag005-B11] Arluck JC , Mayhew AC. Preconception care for the general Ob/Gyn. Clin Obstet Gynecol 2018;61:62–71. 29319589 10.1097/GRF.0000000000000338

[dmag005-B12] Atrash H , Jack BW, Johnson K, Coonrod DV, Moos M, Stubblefield PG, Cefalo R, Damus K, Reddy UM. Where is the “W”oman in MCH? Obstet Gynecol 2008;199:S259–S265. 10.1016/j.ajog.2008.08.059 19081420

[dmag005-B13] Barber A , Flach A, Bonnington J, Pattinson EM. LGBTQ+ healthcare teaching in UK medical schools: an investigation into medical students’ understanding and preparedness for practice. 2023:238212052311648. 10.1177/23821205231164893 PMC10052488 37008793

[dmag005-B14] Benedetto C , Borella F, Divakar H, O’Riordan SL, Mazzoli M, Hanson M, O’Reilly S, Jacobsson B, Conry JA, McAuliffe FM; FIGO Committee on Well Woman Healthcare, FIGO Committee on the Impact of Pregnancy on Long‐Term Health. FIGO preconception checklist: preconception care for mother and baby. Intl J Gynecology & Obste 2024;165:1–8. 10.1002/ijgo.15446 38426290

[dmag005-B15] Berghella V , Buchanan E, Pereira L, Baxter JK. Preconception care. Obstet Gynecol Surv 2010;65:119–131. 20100361 10.1097/OGX.0b013e3181d0c358

[dmag005-B16] Berglund A , Lindmark G. Preconception health and care (PHC)—A strategy for improved maternal and child health. Ups J Med Sci 2016;121:216–221. 27320774 10.1080/03009734.2016.1191564 PMC5098484

[dmag005-B18] Bhutta ZA , Lassi ZS, Bergeron G, Koletzko B, Salam R, Diaz A, McLean M, Black RE, De‐Regil LM, Christian P et al Delivering an action agenda for nutrition interventions addressing adolescent girls and young women: priorities for implementation and research. Ann NY Acad Sci 2017;1393:61–71. 28436103 10.1111/nyas.13352

[dmag005-B19] Bish CL , Farr S, Johnson D, McAnally R. Preconception health of reproductive aged women of the Mississippi River Delta. Matern Child Health J 2012;16:250–257. 23099798 10.1007/s10995-012-1166-9 PMC4536804

[dmag005-B20] Black KI , Dorney E, Hall JA, Pelosi M, Khan SA, Cheney K. Using a validated instrument to assess pregnancy planning and preconception care at antenatal booking visits: a retrospective cohort study. Med J Aust 2023;219:366–370. 37743071 10.5694/mja2.52109

[dmag005-B5399862] Black KZ, , Eng E, , Schaal JC, , Johnson L-S, , Nichols HB, , Ellis KR, , Rowley DL. The Other Side of Through: Young Breast Cancer Survivors' Spectrum of Sexual and Reproductive Health Needs. Qual Health Res 2020;30:2019–2032. 32552407 10.1177/1049732320929649 PMC10557425

[dmag005-B21] Bø K , Artal R, Barakat R, Brown W, Davies GAL, Dooley M, Evenson KR, Haakstad LAH, Henriksson-Larsen K, Kayser B et al Exercise and pregnancy in recreational and elite athletes: 2016 evidence summary from the IOC expert group meeting, Lausanne. Part 1—Exercise in women planning pregnancy and those who are pregnant. Br J Sports Med 2016;50:571–589. 27127296 10.1136/bjsports-2016-096218

[dmag005-B22] Boutain DM , Evans-Agnew R, Liu F, Rosemberg MS. Creating emancipatory dialogues about identity and health by modernizing interviews. ANS Adv Nurs Sci 2018;41:305–315. 30383560 10.1097/ANS.0000000000000233

[dmag005-B23] Boyle JA , Black K, Dorney E, Amor DJ, Brown L, Callander E, Camilleri R, Cheney K, Gordon A, Hammarberg K et al Setting preconception care priorities in Australia using a Delphi technique. Semin Reprod Med 2022;40:214–226. 35760312 10.1055/s-0042-1749683

[dmag005-B24] Braun V , Clarke V. Using thematic analysis in psychology. Qual Res Psychol 2006;3:77–101.

[dmag005-B25] Britton LE , Kaur G, Zork N, Marshall CJ, George M. ‘We tend to prioritise others and forget ourselves’: how women’s caregiving responsibilities can facilitate or impede diabetes self‐management. Diabet Med 2023;40:e15030. 36537593 10.1111/dme.15030 PMC10231690

[dmag005-B26] Brooks C , Supramaniam PR, Mittal M. Preconception health in the well woman. The Obstetric & Gynaecologis 2022;24:58–66.

[dmag005-B27] Broussard DL , Sappenfield WB, Fussman C, Kroelinger CD, Grigorescu V. Core state preconception health indicators: a voluntary, multi-state selection process. Matern Child Health J 2011;15:158–168. 20225127 10.1007/s10995-010-0575-x

[dmag005-B28] Callegari LS , Edmonds SW, Borrero S, Ryan GL, Cusack CM, Zephyrin LC. Preconception care in the veterans health administration. Semin Reprod Med 2018;36:327–339. 31003248 10.1055/s-0039-1678753

[dmag005-B29] Carter T , Schoenaker D, Adams J, Steel A. Paternal preconception modifiable risk factors for adverse pregnancy and offspring outcomes: a review of contemporary evidence from observational studies. BMC Public Health 2023;23:509. 36927694 10.1186/s12889-023-15335-1 PMC10022288

[dmag005-B30] Cassinelli EH , McKinley MC, Kent L, Eastwood K, Schoenaker D, McGowan L. Preconception health and care policies and guidelines in the UK and Ireland: a scoping review. The Lancet 2022;400:S61.

[dmag005-B31] Cassinelli EH , McKinley MC, Kent L, Eastwood K, Schoenaker DAJM, Trew D, Stoikidou T, McGowan L. Preconception health and care policies, strategies and guidelines in the UK and Ireland: a scoping review. 2024; **1**; 1662. 10.1186/s12889-024-19188-0 PMC11193169 38909211

[dmag005-B32] Cheney K , Black K, Pelosi M, Dorney E. Introduction of the London Measure of Unplanned Pregnancy at the booking visit and the midwives’ perspective. BMJ Sex Reprod Health 2023;49:112–117. 10.1136/bmjsrh-2022-201576 36410764

[dmag005-B33] Children’s Alliance, NIHR Southampton Biomedical RC, University of Southampton, University Hospital Southampton NHS FT. A preconception care strategy. 2023.

[dmag005-B34] Chou D , Daelmans B, Jolivet RR, Kinney M, Say L; Every Newborn Action Plan (ENAP) and Ending Preventable Maternal Mortality (EPMM) Working Groups. Ending preventable maternal and newborn mortality and stillbirths. BMJ 2015;351:h4255. 26371222 10.1136/bmj.h4255

[dmag005-B35] City of Toronto. Preconception health information for health professionals. 2024.

[dmag005-B36] Coonrod DV , Bruce NC, Malcolm TD, Drachman D, Frey KA. Knowledge and attitudes regarding preconception care in a predominantly low-income Mexican American population. Am J Obstet Gynecol 2009;200:686.e1–686–e7. 10.1016/j.ajog.2009.02.036 19380123

[dmag005-B37] County of Los Angeles PH. About preconception health. 2001.

[dmag005-B38] Curtis MG. Preconception care: a clinical case of “think globally, act locally”. Am J Obstet Gynecol 2008;199:S257–S258. 19081419 10.1016/j.ajog.2008.07.068

[dmag005-B39] Dean SV , Imam AM, Lassi ZS, Bhutta ZA. Systematic review of preconception risks and interventions. 2013.

[dmag005-B40] Delissaint D , McKyer EL. A systematic review of factors utilized in preconception health behavior research. Health Educ Behav 2011;38:603–616. 21511954 10.1177/1090198110389709

[dmag005-B41] Dennis S. Women’s health: a case for preconception care. 2011.

[dmag005-B42] Dhavliker M , Purohit P. Preconception care: dietary and lifestyle advice. 2017.

[dmag005-B43] Dolan S , Biermann J, Damus K. Genomics for health in preconception and prenatal periods. J Nurs Scholarsh 2007;39:4–9. 17393959 10.1111/j.1547-5069.2007.00136.x

[dmag005-B44] Dorney E , Black KI. Preconception care. Aust J Gen Pract 2018;47:424–429. 30114868 10.31128/AJGP-02-18-4485

[dmag005-B45] Dorney E , Mazza D, Black KI. Interconception care. Aust J Gen Pract 2020;49:317–322. 32464729 10.31128/AJGP-02-20-5242

[dmag005-B46] Dude AM , Schueler K, Schumm LP, Murugesan M, Stulberg DB. Preconception care and severe maternal morbidity in the United States. Am J Obstet Gynecol MFM 2022;4:100549. 34871778 10.1016/j.ajogmf.2021.100549 PMC8891086

[dmag005-B47] Dunlop AL , Jack BW, Bottalico JN, Lu MC, James A, Shellhaas CS, Hallstrom LH, Solomon BD, Feero WG, Menard MK et al The clinical content of preconception care: women with chronic medical conditions. Am J Obstet Gynecol 2008;199:S310–S327. 19081425 10.1016/j.ajog.2008.08.031

[dmag005-B48] East Midlands Maternal Medicine Network, NHS England. Pre-conception care. 2024.

[dmag005-B49] Edmonds SW , Ayres L. Evolutionary concept analysis of reproductive life planning. J Obstet Gynecol Neonatal Nurs 2017;46:78–90. 10.1016/j.jogn.2016.07.012 27837650

[dmag005-B50] Elliott-Mainwaring H. Exploring using NVivo software to facilitate inductive coding for thematic narrative synthesis. Br J Midwifery 2021;29:628–632.

[dmag005-B51] Elmir R , Schmied V. A qualitative study of the impact of adverse birth experiences on fathers. Women Birth 2022;35:e41–e48. 33495131 10.1016/j.wombi.2021.01.005

[dmag005-B52] Global Foundation for the Care of Newborn Infants (GFCNI). Preconception Care – Planning for a Healthy Pregnancy [Internet]. Munich: GFCNI; 2021 [cited 2024 Jun 11]. Available from: https://www.gfcni.org/maternal-newborn-health/maternal-health/preconception-care

[dmag005-B53] Ezegwui HU , Dim C, Dim N, Ikeme AC. Preconception care in South Eastern Nigeria. J Obstet Gynaecol 2008;28:765–768. 19085540 10.1080/01443610802462647

[dmag005-B54] Farahi N , Zolotor A. Recommendations for preconception counseling and care. Am Fam Physician 2013;88:499–506. 24364570

[dmag005-B55] Feldkamp ML , Botto LD. Developing a research and public health agenda for gastroschisis: how do we bridge the gap between what is known and what is not? American J of Med Genetics Pt C 2008;148C:155–161. 10.1002/ajmg.c.30183 18655105

[dmag005-B56] Files JA , David PS, Frey KA. The patient-centered medical home and preconception care: an opportunity for internists. J Gen Intern Med 2008;23:1518–1520. 18506543 10.1007/s11606-008-0657-2 PMC2518012

[dmag005-B57] Files JA , Frey KA, David PS, Hunt KS, Noble BN, Mayer AP. Developing a reproductive life plan. J Midwifery Womens Health 2011;56:468–474. 23181644 10.1111/j.1542-2011.2011.00048.x

[dmag005-B58] Firoz T , McCaw-Binns A, Filippi V, Magee LA, Costa ML, Cecatti JG, Barreix M, Adanu R, Chou D, Say L; the members of the WHO Maternal Morbidity Working Group (MMWG) A framework for healthcare interventions to address maternal morbidity. Int J Gynecol Obstet 2018;141:61–68. 10.1002/ijgo.12469 PMC6001624 29851114

[dmag005-B59] Flenady V , Middleton P, Smith GC, Duke W, Erwich JJ, Khong TY, Neilson J, Ezzati M, Koopmans L, Ellwood D, Lancet’s Stillbirths Series steering committee et al Stillbirths: the way forward in high-income countries. Lancet 2011;377:1703–1717. 21496907 10.1016/S0140-6736(11)60064-0

[dmag005-B60] Floyd RL , Johnson KA, Owens JR, Verbiest S, Moore CA, Boyle C. A national action plan for promoting preconception health and health care in the United States (2012–2014). J Womens Health (Larchmt) 2013;22:797–802. 23944970 10.1089/jwh.2013.4505 PMC4480361

[dmag005-B61] Frayne DJ , Verbiest S, Chelmow D, Clarke H, Dunlop A, Hosmer J, Menard MK, Moos M, Ramos D, Stuebe A et al Health care system measures to advance preconception wellness: consensus recommendations of the clinical workgroup of the national preconception health and health care initiative. Obstet Gynecol 2016;127:863–872. 27054935 10.1097/AOG.0000000000001379

[dmag005-B62] Frey KA. Preconception care by the nonobstetrical provider. Mayo Clin Proc 2002;77:469–473. 12004996 10.4065/77.5.469

[dmag005-B63] Frey KA , Navarro SM, Kotelchuck M, Lu MC. The clinical content of preconception care: preconception care for men. Am J Obstet Gynecol 2008;199:S389–S395. 19081435 10.1016/j.ajog.2008.10.024

[dmag005-B64] Gavin L , Moskosky S, Carter M, Curtis K, Glass E, Godfrey E, Marcell A, Mautone-Smith N, Pazol K, Tepper N et al Providing quality family planning services: recommendations of CDC and the U.S. Office of Population Affairs. 2014;04:1–54. 24759690

[dmag005-B65] Government of Belize, Ministry of Health. Preconception care and obstetrics primary health care continuum of care. 2018.

[dmag005-B66] Government of Canada. Preconception health: health before pregnancy. 2023.

[dmag005-B67] Gregory EF , Upadhya KK, Cheng TL, Psoter KJ, Mistry KB. Enabling factors associated with receipt of interconception health care. Matern Child Health J 2020;24:275–282. 31838666 10.1007/s10995-019-02850-0 PMC7117827

[dmag005-B68] Guler Baysoy N , Özkan S. Preconception care: a public health perspective. 2012:77–90.

[dmag005-B69] Hall J , Chawla M, Watson D, Jacob CM, Schoenaker D, Connolly A, Barrett G, Stephenson J. Addressing reproductive health needs across the life course: an integrated, community-based model combining contraception and preconception care. Lancet Public Health 2023;8:e76–e84. 36603914 10.1016/S2468-2667(22)00254-7

[dmag005-B70] Harris NL , Richardson Cayama M, Arias C, Ansari F, Ilonzo C, Williams A, Sappenfield W, Kirby RS. Assessing the unmet preconception care needs of men in the United States by race/ethnicity and nativity. Sex Reprod Healthc 2023;36:100840. 37001422 10.1016/j.srhc.2023.100840

[dmag005-B71] Harville EW , Mishra GD, Yeung E, Mumford SL, Schisterman EF, Jukic AM, Hatch EE, Mikkelsen EM, Jiang H, Ehrenthal DB et al The Preconception Period analysis of Risks and Exposures Influencing health and Development (PrePARED) consortium. Paediatr Perinat Epidemiol 2019;33:490–502. 31659792 10.1111/ppe.12592 PMC6901022

[dmag005-B72] Hemsing N , Greaves L, Poole N. Preconception health care interventions: a scoping review. Sex Reprod Healthc 2017;14:24–32. 29195631 10.1016/j.srhc.2017.08.004

[dmag005-B73] Hill B , Hall J, Skouteris H, Currie S. Defining preconception: exploring the concept of a preconception population. BMC Pregnancy Childbirth 2020;20:280. 32381056 10.1186/s12884-020-02973-1 PMC7206804

[dmag005-B74] Howard L , Easter A, Atmore K. Delivering preconception care to women of childbearing age with serious mental illness. 2020.

[dmag005-B75] Huberty J , Leiferman JA, Kruper AR, Jacobson LT, Waring ME, Matthews JL, Wischenka DM, Braxter B, Kornfield SL. Exploring the need for interventions to manage weight and stress during interconception. J Behav Med 2017;40:145–158. 27858206 10.1007/s10865-016-9813-z PMC5358329

[dmag005-B76] Iseyemi A , Zhao Q, McNicholas C, Peipert JF. Socioeconomic status as a risk factor for unintended pregnancy in the contraceptive CHOICE project. Obstet Gynecol 2017;130:609–615. 28796678 10.1097/AOG.0000000000002189 PMC5654472

[dmag005-B77] Jack B , Bickmore T, Hempstead M, Yinusa-Nyahkoon L, Sadikova E, Mitchell S, Gardiner P, Adigun F, Penti B, Schulman D et al Reducing preconception risks among African American women with conversational agent technology. J Am Board Fam Med 2015;28:441–451. 26152434 10.3122/jabfm.2015.04.140327 PMC4739811

[dmag005-B78] Jack BW , Atrash H, Coonrod DV, Moos M, O’Donnell J, Johnson K. The clinical content of preconception care: an overview and preparation of this supplement. Am J Obstet Gynecol 2008;199:S266–S279. 19081421 10.1016/j.ajog.2008.07.067

[dmag005-B80] Jacob CM , Hanson M. The preconception period as a platform for preventing diabetes and non‐communicable diseases. Practical Diabetes 2022;39:14–18.

[dmag005-B81] Jacob CM , Newell M, Hanson M. Narrative review of reviews of preconception interventions to prevent an increased risk of obesity and non‐communicable diseases in children. Obes Rev 2019;20:5–17. 31419048 10.1111/obr.12769

[dmag005-B82] Johnson KA , Gee RE. Interpregnancy care. Semin Perinatol 2015;39:310–315. 26188595 10.1053/j.semperi.2015.05.011

[dmag005-B83] Johnson K , Atrash H, Johnson A. Policy and finance for preconception care. Womens Health Issues 2008;18:S2–S9. 19059547 10.1016/j.whi.2008.09.006

[dmag005-B84] Johnson K , Posner SF, Biermann J, Cordero JF, Atrash HK, Parker CS, Boulet S, Curtis MG. Recommendations to improve preconception health and Health Care—United States: report of the CDC/ATSDR preconception care work group and the select panel on preconception care. 2006;6:1. CE. 16617292

[dmag005-B85] Khekade H , Potdukhe A, Taksande AB, Wanjari MB, Yelne S. Preconception care: a strategic intervention for the prevention of neonatal and birth disorders. Cureus 2023;15:e41141. 37519532 10.7759/cureus.41141 PMC10386873

[dmag005-B86] Kimport K. More than a physical burden: women’s mental and emotional work in preventing pregnancy. J Sex Res 2018;55:1096–1105. 28418714 10.1080/00224499.2017.1311834 PMC6115298

[dmag005-B87] Kizirian NV , Black KI, Musgrave L, Hespe C, Gordon A. Understanding and provision of preconception care by general practitioners. Aust N Z J Obstet Gynaecol 2019;59:799–804. 30773610 10.1111/ajo.12962

[dmag005-B88] Kotelchuck M , Lu M. Father’s role in preconception health. Matern Child Health J 2017;21:2025–2039. 28983715 10.1007/s10995-017-2370-4

[dmag005-B89] Lancet. Campaigning for preconception health. The Lancet 2018;10132:1749. 10.1016/S0140-6736(18)30981-4 29739547

[dmag005-B90] Lassi ZS , Dean SV, Mallick D, Bhutta ZA. Preconception care: delivery strategies and packages for care. Reprod Health 2014;11:S7. 25415178 10.1186/1742-4755-11-S3-S7 PMC4196568

[dmag005-B91] Liu F. Reproductive health policy in China: a study of preconception care in rural China. IJWHR 2015;3:13–20.

[dmag005-B92] Lu MC , Kotelchuck M, Culhane JF, Hobel CJ, Klerman LV, Thorp JM. Preconception care between pregnancies: the content of internatal care. Matern Child Health J 2006;10:S107–122. 16817001 10.1007/s10995-006-0118-7 PMC1592148

[dmag005-B93] Malcom X. The difference between preconception and prenatal care. 2018.

[dmag005-B94] Manchester MU. The reasons why good pre-conception care is important. 2024.

[dmag005-B95] March of Dimes. Getting ready for pregnancy: preconception health. 2020.

[dmag005-B96] Marcin A. Preconception and what it means for pregnancy. 2024.

[dmag005-B97] Margerison CE , Kaestner R, Chen J, MacCallum-Bridges C. Impacts of medicaid expansion before conception on prepregnancy health, pregnancy health, and outcomes. Am J Epidemiol 2021;190:1488–1498. 33423053 10.1093/aje/kwaa289 PMC8522774

[dmag005-B98] Marshall C , Britton LE. Pre-pregnancy care for women with diabetes is critical, but inadequately delivered. 2020.

[dmag005-B99] Mason E , Chandra-Mouli V, Baltag V, Christiansen C, Lassi ZS, Bhutta ZA. Preconception care: advancing from ‘important to do and can be done’ to ‘is being done and is making a difference’. Reprod Health 2014;11:S8. 25415261 10.1186/1742-4755-11-S3-S8 PMC4196570

[dmag005-B100] Mazza D , Chapman A, Michie S. Barriers to the implementation of preconception care guidelines as perceived by general practitioners: a qualitative study. BMC Health Serv Res 2013;13:36. 23368720 10.1186/1472-6963-13-36 PMC3565953

[dmag005-B101] Mello S , Tan ASL, Sanders-Jackson A, Bigman CA. Gender stereotypes and preconception health: men’s and women’s expectations of responsibility and intentions to engage in preventive behaviors. Matern Child Health J 2019;23:459–469. 30552600 10.1007/s10995-018-2654-3

[dmag005-B102] Methodist. Planning for pregnancy: why preconception health is important for women and men. 2021.

[dmag005-B103] Monson M , Jackson M. Pregnancy after bariatric surgery. Clin Obstet Gynecol 2016;59:158–171. 26710306 10.1097/GRF.0000000000000178

[dmag005-B104] Montanaro C , Robson L, Binnington L, Winters N, Brown HK. Validating PreCHAT: a digital preconception health risk assessment tool to improve reproductive, maternal and child health. Can J Nurs Res 2023;55:206–215. 35816292 10.1177/08445621221112668

[dmag005-B105] Moos M. From Concept to practice: reflections on the preconception health agenda. J Womens Health (Larchmt) 2010;19:561–567. 20184531 10.1089/jwh.2009.1411

[dmag005-B106] Munthali M , Chiumia IK, Mandiwa C, Mwale S. Knowledge and perceptions of preconception care among health workers and women of reproductive age in Mzuzu City, Malawi: a cross-sectional study. Reprod Health 2021;18:229. 34775983 10.1186/s12978-021-01282-w PMC8591898

[dmag005-B107] Murugappan G , Li S, Leonard SA, Winn VD, Druzin ML, Eisenberg ML. Association of preconception paternal health and adverse maternal outcomes among healthy mothers. Am J Obstet Gynecol MFM 2021;3:100384. 33895399 10.1016/j.ajogmf.2021.100384 PMC13221126

[dmag005-B108] National Institute for Health and Clinical Excellence. Pre-conception: advice and management: what is it? 2023.

[dmag005-B109] National Library of Medicine. Preconception care. 2016. 10.1080/15360280801989377 28792816

[dmag005-B110] New Road Medical Centre, N.H.S. Pre-conception health. 2021.

[dmag005-B111] New York State Department of Health. Preconception care: a guide for optimizing outcomes. 2013.

[dmag005-B112] Nikolaou D. What is preconception care? 2023.

[dmag005-B113] Nobles‐Botkin J , Lincoln A, Cline J. Preconception care resources: where to start. J Midwife Womens Health 2016;61:365–369. 10.1111/jmwh.12464 27151890

[dmag005-B114] Nugent CN , Chandra A. Infertility and impaired fecundity in women and men in the United States, 2015–2019. Natl Health Stat Report 2024;1. 38722687

[dmag005-B115] Nypaver C , Arbour M, Niederegger E. Preconception care: improving the health of women and families. J Midwifery Womens Health 2016;61:356–364. 27218593 10.1111/jmwh.12465

[dmag005-B116] O’Connor H , Willcox JC, De Jersey S, Wright C, Wilkinson SA. Digital preconception interventions targeting weight, diet and physical activity: a systematic review. Nutr Diet 2024;81:244–260. 37845187 10.1111/1747-0080.12842

[dmag005-B117] Ojifinni OO , Ibisomi L. Preconception care practices in Nigeria: a descriptive qualitative study. Reprod Health 2020;17:172. 33148313 10.1186/s12978-020-01030-6 PMC7640668

[dmag005-B118] Okemo J , Temmerman M, Mwaniki M, Kamya D. Preconception care among pregnant women in an urban and a rural health facility in Kenya: a quantitative study. 2020;20. 10.3390/ijerph17207430 PMC7601657 33065989

[dmag005-B119] Ontario Public Health Association. Shift-Enhancing the health of Ontarians: a call to action for preconception health promotion and care. 2014.

[dmag005-B120] Paladine HL , Ekanadham H, Diaz DC. Health maintenance for women of reproductive age. Am Fam Physician 2021;103:209–217. 33587575

[dmag005-B121] Pentecost M , Meloni M. “It’s never too early”: preconception care and postgenomic models of life. Front Sociol 2020;5:21. 33869430 10.3389/fsoc.2020.00021 PMC8022598

[dmag005-B122] Permezel J , Arnold ASC, Thomas J, Maepioh AL, Brown R, Hafford-Letchfield T, Skouteris H, Hatzikiriakidis K, McNair RP. Experiences in the delivery of preconception and pregnancy care for LGBTIQA+ people: a systematic review and thematic synthesis of patient and healthcare provider perspectives. Midwifery 2023;123:103712. 37178659 10.1016/j.midw.2023.103712

[dmag005-B123] Peterson C , Grosse SD, Li R, Sharma AJ, Razzaghi H, Herman WH, Gilboa SM. Preventable health and cost burden of adverse birth outcomes associated with pregestational diabetes in the United States. Am J Obstet Gynecol 2015;212:74.e1–74–e9. 10.1016/j.ajog.2014.09.009 PMC4469071 25439811

[dmag005-B124] Planned Parenthood. Pre-pregnancy health and planning. 2024.

[dmag005-B125] Poels M , Koster MPH, Franx A, van Stel HF. Parental perspectives on the awareness and delivery of preconception care. BMC Pregnancy Childbirth 2017;17:324. 28950838 10.1186/s12884-017-1531-1 PMC5615801

[dmag005-B126] Popay J , Roberts H, Sowden A, Petticrew M, Arai L, Rodgers M, Britten N, Roen K, Duffy S. Guidance on the conduct of narrative synthesis in systematic reviews: a product from the ESRC Methods Programme. 2006.

[dmag005-B127] Posner SF , Johnson K, Parker C, Atrash H, Biermann J. The national summit on preconception care: a summary of concepts and recommendations. Matern Child Health J 2006;10:S197–S205. 16773451 10.1007/s10995-006-0107-x PMC1592248

[dmag005-B128] Price SA , Sumithran P, Nankervis A, Permezel M, Proietto J. Preconception management of women with obesity: a systematic review. Obes Rev 2019;20:510–526. 30549166 10.1111/obr.12804

[dmag005-B129] Public Health Agency of Canada. Family-Centred Maternity and Newborn Care. National Guidelines, 2017.

[dmag005-B130] Public Health England. Making the case for preconception care. 2018.

[dmag005-B131] Raynal P. La consultation préconceptionnelle. Gynécologie Obstétrique & Fertilité 2010;38:481–485. 10.1016/j.gyobfe.2010.05.015 20576457

[dmag005-B132] Reproductive Health National TC. Preconception health toolkit. 2022.

[dmag005-B133] Righton O , Flynn A, Alwan NA, Schoenaker D. Preconception health in adolescence and adulthood across generations in the UK: findings from three British birth cohort studies. PLoS One 2024;19:e0299061. 39661574 10.1371/journal.pone.0299061 PMC11633974

[dmag005-B134] Robbins CL , Gavin L, Carter MW, Moskosky SB. The link between reproductive life plan assessment and provision of preconception care at publicly funded health centers. Perspect Sex Reprod Health 2017;49:167–172. 28475825 10.1363/psrh.12030 PMC5603190

[dmag005-B135] Rosenberger KD , Cibulka NJ, Barron ML. Guidelines for Nurse Practitioners in Ambulatory Obstetric Settings. New York, NY: Springer Publishing Company, 2022.

[dmag005-B160] Royal Australian and New Zealand College of Obstetricians and Gynaecologists (RANZCOG). Pre-pregnancy counselling (C-Obs 3a) [Internet]. Melbourne: RANZCOG; 2024 [cited 2024 May 20]. Available from: https://ranzcog.edu.au/wp-content/uploads/2022/05/Pre-pregnancy-Counselling.pdf (20 May 2024, date last accessed).

[dmag005-B136] Rural Health Information Hub. Models Addressing Preconception Health. 2024.

[dmag005-B137] Salihu HM , Myers J, August EM. Pregnancy in the workplace. Occup Med (Lond) 2012;62:88–97. 22355087 10.1093/occmed/kqr198

[dmag005-B138] Sanders LB. Preconception care: practice and policy implications for nurses. Policy Polit Nurs Pract 2009;10:129–133. 19783535 10.1177/1527154409338494

[dmag005-B139] Santos BNSD , Araújo FG, Paula TFD, Matozinhos FP, Felisbino-Mendes M. Prevalence of preconception health indicators among Brazilian women of reproductive age. Cien Saude Colet 2023;28:3367–3381. 37971017 10.1590/1413-812320232811.16282022

[dmag005-B140] Sardasht FG , Motaghi Z, Keramat A, Shariati M, Akbari N. Women’s and care providers’ perspectives of quality preconception care: a qualitative descriptive study. Iran J Nurs Midwifery Res 2022;27:337–345. 36275333 10.4103/ijnmr.ijnmr_260_20 PMC9580574

[dmag005-B141] Schoenaker D , Gafari O, Taylor E, Hall J, Barker C, Jones B, Alwan NA, Watson D, Jacob CM, Barker M et al What does ‘preconception health’. Mean to People? A Public Consultation on Awareness and Use of Language 2024a;4:e14181. 10.1111/hex.14181 PMC11344208 39180340

[dmag005-B142] Schoenaker D , Lovegrove E, Santer M, Matvienko-Sikar K, Carr H, Alwan NA, Kubelabo L, Davies N, Godfrey KM. Developing consensus on priorities for preconception care in the general practice setting in the UK: study protocol. PLoS One 2024b;19:e0311578. 39570956 10.1371/journal.pone.0311578 PMC11581211

[dmag005-B143] Schoonen M , Van Der Zee B, Wildschut H, De Beaufort I, De Wert G, De Koning H, Essink‐Bot M, Steegers E. Informing on prenatal screening for Down syndrome prior to conception. An empirical and ethical perspective. Am J Med Genet A 2012;158A:485–497. 22302760 10.1002/ajmg.a.35213

[dmag005-B144] Shangaris P. The vital role of preconception care in ensuring healthy pregnancies. 2023.

[dmag005-B145] Shannon G , Alberg C, Nacul L, Pashayan N. Preconception health care and congenital disorders: mathematical modelling of the impact of a preconception care programme on congenital disorders. BJOG 2013;120:555–566. 23331865 10.1111/1471-0528.12116

[dmag005-B146] Sijpkens MK , Van Den Hazel CZ, Delbaere I, Tydén T, Mogilevkina I, Steegers EAP, Shawe J, Rosman AN. Results of a Dutch national and subsequent international expert meeting on interconception care. J Matern Fetal Neonatal Med 2020;33:2232–2240. 30606078 10.1080/14767058.2018.1547375

[dmag005-B147] Sijpkens MK , Van Voorst SF, Rosman AN, De Jong-Potjer LC, Denktaş S, Koch BCP, Bertens LCM, Steegers EAP. Change in lifestyle behaviors after preconception care: a prospective cohort study. Am J Health Promot 2021;35:116–120. 32431156 10.1177/0890117120927287 PMC7747029

[dmag005-B148] Simone J , Hoyt MJ, Storm DS, Finocchario-Kessler S. Models of HIV preconception care and key elements influencing these services: findings from healthcare providers in seven US cities. AIDS Patient Care STDS 2018;32:272–281. 29870269 10.1089/apc.2017.0299 PMC6034389

[dmag005-B149] St. Fleur M , Damus K, Jack B. The future of preconception care in the United States: multigenerational impact on reproductive outcomes. Ups J Med Sci 2016;121:211–215. 27434227 10.1080/03009734.2016.1206152 PMC5098483

[dmag005-B150] Stanhope KK , Kramer MR. Association between recommended preconception health behaviors and screenings and improvements in cardiometabolic outcomes of pregnancy. 2021. 10.5888/pcd18.200481 PMC7845551 33476258

[dmag005-B151] State of Rhode Island Department, of Health. Health information for women planning a pregnancy. 2024.

[dmag005-B152] Steegers EAP. Understanding preconception health for early life course medicine. Paediatr Perinat Epidemiol 2019;33:503–505. 31637731 10.1111/ppe.12597

[dmag005-B153] Steel A , Lucke J, Reid R, Adams J. A systematic review of women’s and health professional’s attitudes and experience of preconception care service delivery. Fam Pract 2016;33:588–595. 27650308 10.1093/fampra/cmw094

[dmag005-B154] Stephenson J , Heslehurst N, Hall J, Schoenaker DAJM, Hutchinson J, Cade JE, Poston L, Barrett G, Crozier SR, Barker M et al Before the beginning: nutrition and lifestyle in the preconception period and its importance for future health. Lancet 2018;391:1830–1841. 29673873 10.1016/S0140-6736(18)30311-8 PMC6075697

[dmag005-B155] Stroumsa D , Johnson TRB. Improving preconception health among sexual minority women. J Womens Health (Larchmt) 2020;29:745–747. 32096677 10.1089/jwh.2020.8319

[dmag005-B156] Taher SWb. Pre Pregnancy Care (PPC). 2019.

[dmag005-B157] Tarasoff LA , Lunsky Y, Chen S, Guttmann A, Havercamp SM, Parish SL, Vigod SN, Carty A, Brown HK. Preconception health characteristics of women with disabilities in Ontario: a population-based, cross-sectional study. J Womens Health (Larchmt) 2020;29:1564–1575. 32678692 10.1089/jwh.2019.8273 PMC7757535

[dmag005-B158] Temel S , Van Voorst SF, De Jong-Potjer LC, Waelput AJM, Cornel MC, De Weerd SR, Denktaş S, Steegers EAP. The Dutch national summit on preconception care: a summary of definitions, evidence and recommendations. J Community Genet 2015;6:107–115. 25394755 10.1007/s12687-014-0204-2 PMC4286565

[dmag005-B159] Temel S , Van Voorst SF, Jack BW, Denktaş S, Steegers EAP. Evidence-based preconceptional lifestyle interventions. Epidemiol Rev 2014;36:19–30. 23985430 10.1093/epirev/mxt003

[dmag005-B161] The Surveillance and Research Workgroup and Clinical Workgroup of the National Preconception Health and Health, Care Initiative, Adamski A, Bernstein PS, Boulet SL, Chowdhury FM, D’Angelo DV, Coonrod DV, Frayne DJ, Kroelinger C, Morgan IA et al Surveillance Indicators for Women’s Preconception Care 2020;7:910–918. 10.1089/jwh.2019.8146 PMC7991894 32357078

[dmag005-B162] Thompson EL , Vázquez-Otero C, Vamos CA, Marhefka SL, Kline NS, Daley EM. Rethinking preconception care: a critical, women’s health perspective. Matern Child Health J 2017;21:1147–1155. 28078529 10.1007/s10995-016-2213-8

[dmag005-B163] Tijoriwala S. FIGO Preconception checklist: a valuable new tool to address health risks during the preconception period. 2024.

[dmag005-B164] Tufford L , Newman P. Bracketing in qualitative research. Qualitative Social Work 2012;11:80–96.

[dmag005-B165] Turnbull D , Chugh R, Luck J. Systematic-narrative hybrid literature review: a strategy for integrating a concise methodology into a manuscript. Social Sciences & Humanities Open 2023;7:100381.

[dmag005-B166] Tydén T. Why is preconception health and care important? Ups J Med Sci 2016;121:207. 27487464 10.1080/03009734.2016.1211776 PMC5098481

[dmag005-B167] UK Parliament. Preconception care strategy. 2023.

[dmag005-B168] University of Monash. Pre-pregnancy risk factors are common amongst women of reproductive age. 2024.

[dmag005-B169] Usman T , Mullins E. Pregnancy planning and preconception health in a pandemic: a patient and public involvement (PPI) qualitative study. 2021:; 4–144.

[dmag005-B170] Van Der Zee B , De Beaufort I. Preconception care: a parenting protocol. A moral inquiry into the responsibilities of future parents towards their future children. Bioethics 2011;25:451–457. 21929704 10.1111/j.1467-8519.2011.01924.x

[dmag005-B171] Van Der Zee B , De Beaufort ID, Steegers EAP, Denktas S. Perceptions of preconception counselling among women planning a pregnancy: a qualitative study. Fam Pract 2013;30:341–346. 23180815 10.1093/fampra/cms074

[dmag005-B172] Van Der Zee B , De Beaufort I, Temel S, De Wert G, Denktas S, Steegers E. Preconception care: an essential preventive strategy to improve children’s and women’s health. J Public Health Policy 2011;32:367–379. 21808249 10.1057/jphp.2011.13

[dmag005-B173] Van Voorst SF , Ten Kate CA, De Jong‐Potjer LC, Steegers EAP, Denktaş S. Developing social marketed individual preconception care consultations: which consumer preferences should it meet? Health Expect 2017;20:1106–1113. 28440578 10.1111/hex.12555 PMC5600215

[dmag005-B174] Verbiest S , Yates L, Neely EJ, Tumblin C. Looking back, visioning forward: preconception health in the US 2005 to 2023. Matern Child Health J 2025;29:1123–1137. 37864771 10.1007/s10995-023-03788-0

[dmag005-B175] Vink-van Os LCA , Birnie E, van Vliet-Lachotzki EH, Bonsel GJ, Steegers EAP. Determining pre-conception risk profiles using a national online self-reported risk assessment: a cross-sectional study. Public Health Genomics 2015;18:204–215. 25967756 10.1159/000381449

[dmag005-B176] Waggoner MR. Motherhood preconceived: the emergence of the preconception health and health care initiative. J Health Polit Policy Law 2013;38:345–371. 23262764 10.1215/03616878-1966333 PMC3656594

[dmag005-B177] Wallace M , Hurwitz B. Preconception care: who needs it, who wants it, and how should it be provided? Br J Gen Pract 1998;48:963–966. 9624765 PMC1409978

[dmag005-B178] Watkins A. Preconception Care. 2023.

[dmag005-B179] Watterson C , MacDonald-Wicks L, Collins C, Hutchesson M, Shrewsbury V, Vincze L, Heslehurst N, Follong B. Effectiveness of maternal dietary interventions for improving mother and infant health outcomes: an umbrella review protocol. JBI Database System Rev Implement Rep 2018;16:1929–1938. 10.11124/JBISRIR-2017-003653 30335039

[dmag005-B180] Welshman H , Dombrowski S, Grant A, Swanson V, Goudreau A, Currie S. Preconception knowledge, beliefs and behaviours among people of reproductive age: a systematic review of qualitative studies. Prev Med 2023;175:107707. 37730135 10.1016/j.ypmed.2023.107707

[dmag005-B181] West of Scotland Managed Clinical Network for Sexual Health Clinical Guidelines Group. What Is Preconception Health? 2021.

[dmag005-B182] Wilson C , Howard LM, Reynolds RM, Simonoff E, Ismail K. Preconception health. Lancet 2018;392:2266–2267. 10.1016/S0140-6736(18)32199-8 30496117

[dmag005-B183] Withanage N , Botfield J, Mazza D. Family planning: the importance of preconception health. 2022.

[dmag005-B184] World Health Organization. Preconception care to reduce maternal and childhood mortality and morbidity. 2012.

[dmag005-B185] World Health Organization. Preconception care: regional expert group consultation. 2013a.

[dmag005-B186] World Health Organization. Preconception care: maximising the gains for maternal and child health—Policy brief World Health Organization. 2013b.

[dmag005-B187] World Health Organization. Maternal mortality. 2024.

[dmag005-B188] Xaverius PK , Tenkku LE, Salas J. Differences between women at higher and lower risk for an unintended pregnancy. Womens Health Issues 2009;19:306–312. 19733800 10.1016/j.whi.2009.06.002

[dmag005-B189] Young CT , Urquia ML, Ray JG. Preconception care in low- and middle-income countries: new opportunities and a new metric. PLoS Med 2013;10:e1001507. 24019761 10.1371/journal.pmed.1001507 PMC3760777

[dmag005-B190] Zaçe D , Orfino A, Viteritti AM, Versace V, Di Pietro ML, Ricciardi WA. Comprehensive assessment of preconception health needs and interventions regarding women in childbearing age: a systematic review. 2022:; E174–Pages. 10.15167/2421-4248/jpmh2022.63.1.2391 PMC9121675 35647378

[dmag005-B191] Zühlke L , Acquah L. Pre-conception counselling for key cardiovascular conditions in Africa: optimising pregnancy outcomes. Cardiovasc J Afr 2016;27:79–83. 27213854 10.5830/CVJA-2016-017 PMC4928169

